# The efficacy and safety of intrathecal pemetrexed for leptomeningeal metastasis from non-small cell lung cancer: a single-arm meta-analysis of Chinese patients

**DOI:** 10.3389/fonc.2025.1543416

**Published:** 2025-06-18

**Authors:** Yushi Zhao, Xueqin Gao, Yong Han

**Affiliations:** Department of Pharmacy, Union Hospital, Tongji Medical College, Huazhong University of Science and Technology, Wuhan, China

**Keywords:** pemetrexed, non-small cell lung cancer, leptomeningeal metastasis, meta-analysis, efficacy, safety

## Abstract

**Objectives:**

The purpose of this meta-analysis is to evaluate the safety and effectiveness of intrathecal pemetrexed (IP) in patients with non-small cell lung cancer with leptomeningeal metastasis (NSCLC-LM).

**Methods:**

A systematic search of PubMed, the Cochrane library, Embase, and ClinicalTrials.gov databases was executed until December 11, 2024. The quality of the selected studies was evaluated using the Risk Of Bias In Non-randomized Studies Of Interventions (ROBINS-I) tool. Data extracted encompassed disease control rate (DCR), objective response rate (ORR), median overall survival (mOS), and adverse events (AEs). A random-effects model was used in the meta-analysis, which was conducted using STATA 15.1 software. Egger’s and Begg’s tests were used to analyze publication bias, and when significant publication bias was detected, the Trim and Fill method was employed to adjust for the bias.

**Results:**

This meta-analysis included 8 studies involving a total of 306 patients, with a pooled ORR of 57.6% (95% CI: 39.5%-74.7%). Further subgroup analysis revealed that a pemetrexed dosage of 40–50 mg exhibited superior efficacy, with an ORR of 84.5% (95% CI: 70.0%-95.6%), compared to an ORR of 46.6% (95% CI: 29.2%-64.4%) for dosages of 10–30 mg. Additionally, patients with EGFR mutations exhibited an ORR of 56.2% (95% CI: 34.7%-76.6%), while those with other genetic subtypes had an ORR of 44.8% (95% CI: 25.1%-65.1%). The combined DCR was 85.4% (95% CI: 76.5%-92.7). In terms of survival, the pooled data from 6 studies yielded a mOS of 8.12 months (95% CI: 6.07-10.17). Common adverse events associated with pemetrexed included myelosuppression (32.6%), headache (24.8%), abnormal transaminase (11.8%), nausea (7.3%), vomiting (11.7%), radiculitis (8.4%) and leukoencephalopathy (6.4%). Potential publication bias was identified for DCR and grade≥III myelosuppression. Subgroup analyses performed by DCR showed that the bias was related to drug dosage, while the Trim and Fill method for grade ≥III myelosuppression did not reverse the bias. These findings suggest that publication bias had minimal impact and that the results were relatively stable.

**Conclusions:**

This meta-analysis concludes that patients with NSCLC-LM benefit from intrathecal chemotherapy using pemetrexed.

## 1 Introduction

Leptomeningeal metastasis (LM) refers to the dissemination of malignant tumor cells into the leptomeninges, subarachnoid space and other regions of the cerebrospinal fluid (CSF) (1). LM is a serious complication, occurring in approximately 3%-5% of patients with non-small cell lung cancer (NSCLC), and is associated with an extremely poor prognosis (2). The primary challenge in the treatment of LM lies in overcoming the blood-brain barrier (BBB) to achieve effective drug concentrations in the CSF. Pemetrexed, a multi-targeted anti-folate metabolic drug, when administered through intrathecal injection, directly enters the CSF, bypassing the BBB, thereby effectively eliminating tumor cells attached to the leptomeninges (3). Previously, the main drugs for IC have included methotrexate (MTX), cytarabine (Ara-c), and thiotepa. However, their efficacy in treating LM has been constrained (4–7). Therefore, there is an urgent need for developing novel therapy strategies to enhance the long-term management of LM in NSCLC patients.

Pemetrexed, a multi-targeted antifolate agent, inhibits key enzymes involved in folate metabolism and exhibits potent antitumor activity. Unlike MTX, pemetrexed targets multiple pathways, including dihydrofolate reductase and glycinamide ribonucleotide formyltransferase, exerting a broader antitumor effect (8). As a first-line treatment for NSCLC, especially in patients with non-squamous histology, pemetrexed has demonstrated excellent efficacy and cytotoxicity. Studies have shown that patients receiving pemetrexed maintenance therapy without central nervous system (CNS) metastasis experience a lower incidence of CNS metastases compared to those on alternative regimens (9). The use of intrathecal pemetrexed offers a novel treatment method for patients with NSCLC-LM, potentially controlling leptomeningeal lesions and prolonging survival. However, current evidence on intrathecal pemetrexed for LM is primarily derived from non-randomized studies with small sample sizes, limiting the robustness of efficacy and safety assessments. Thus, we conducted a meta-analysis to evaluate the safety and efficacy of intrathecal pemetrexed in patients with NSCLC-LM, aiming to provide evidence-based insights and expand treatment options for clinical management of this challenging condition.

## 2 Methods

### 2.1 Retrieval strategy

A systematic search was conducted across four databases: PubMed, the Cochrane library, Embase, and ClinicalTrials.gov. The final search was performed on December 11, 2024, using the following MeSH terms and free-text keywords: “Pemetrexed OR Alimta OR LY 231514 OR MTA” and “CNS OR leptomeningeal metastasis OR carcinomatous meningitis OR leptomeningeal metastases”. Only English-language studies were included in the search. The specific search strategy is referenced in **Supplementary Table S1**.

### 2.2 Selection criteria

The includes studies that fulfilled the following criteria: (1) Population: patients diagnosed with NSCLC-LM; (2) Intervention: intrathecal pemetrexed, either as monotherapy or in combination with other chemotherapeutic drugs; (3) Study type: prospective and retrospective studies, including randomized controlled trials, cohort studies, and single-arm studies; (4) Outcome measures: Studies reporting outcomes: objective response rate (ORR), disease control rate (DCR), overall survival (OS), or adverse events (AEs).

Exclusion criteria: *in vitro* experiments, animal investigations, literature reviews, meta-analyses, duplicate publications, case reports and letters.

### 2.3 Data extraction and quality assessment

Two investigators independently extracted data from all studies, and any disagreements were resolved via joint discussion with a third researcher. Subsequently, the quality of the studies was assessed. Extracted data included the following: author name, year of publication, intervention, reported outcome measures and so on. Clinical and safety endpoints comprised ORR, DCR, OS, incidence of major adverse events, and incidence of grade ≥3 adverse events.

Furthermore, the quality of the included non-randomized studies was assessed using the Risk Of Bias In Non-randomized Studies-of Interventions (ROBINS-I) tool (10). The ROBINS-I tool evaluates bias across seven domains. Each domain was rated based on responses to signaling questions (“Yes”, “Probably Yes”, “Probably No”, “No” and “No Information”) with the overall risk of bias categorized as low, moderate, serious, critical or no information.

### 2.4 Statistical analysis

Meta-analyses were carried out using the metaprop program in Stata software (version 15.1), which is designed for binomial data analysis. This method accurately calculates binomial proportions and confidence intervals based on test scores (11). Statistical heterogeneity was assessed by I^2^ and Cochran’s Q test, with I^2^ values >50% or P values ≤0.1 indicating significant heterogeneity. Given that previous single-arm meta-analyses have reported I^2^ generally exceeding 90% (12), random-effects models, which are more robust in the presence of high heterogeneity (13), were used in this study regardless of heterogeneity levels. Egger’s and Begg’s tests were used to quantify and visually represent publication bias, with P < 0.05 signifying significant bias. Furthermore, sensitivity analyses were conducted to assess the stability of the results. When publication bias was identified, its effects were evaluated using the Trim and Fill method. If the impact was not significant, the results were considered reliable and stable, and if the impact was significant, it was fully discussed before drawing final conclusions.

## 3 Result

### 3.1 Study selection

**Figure 1:**
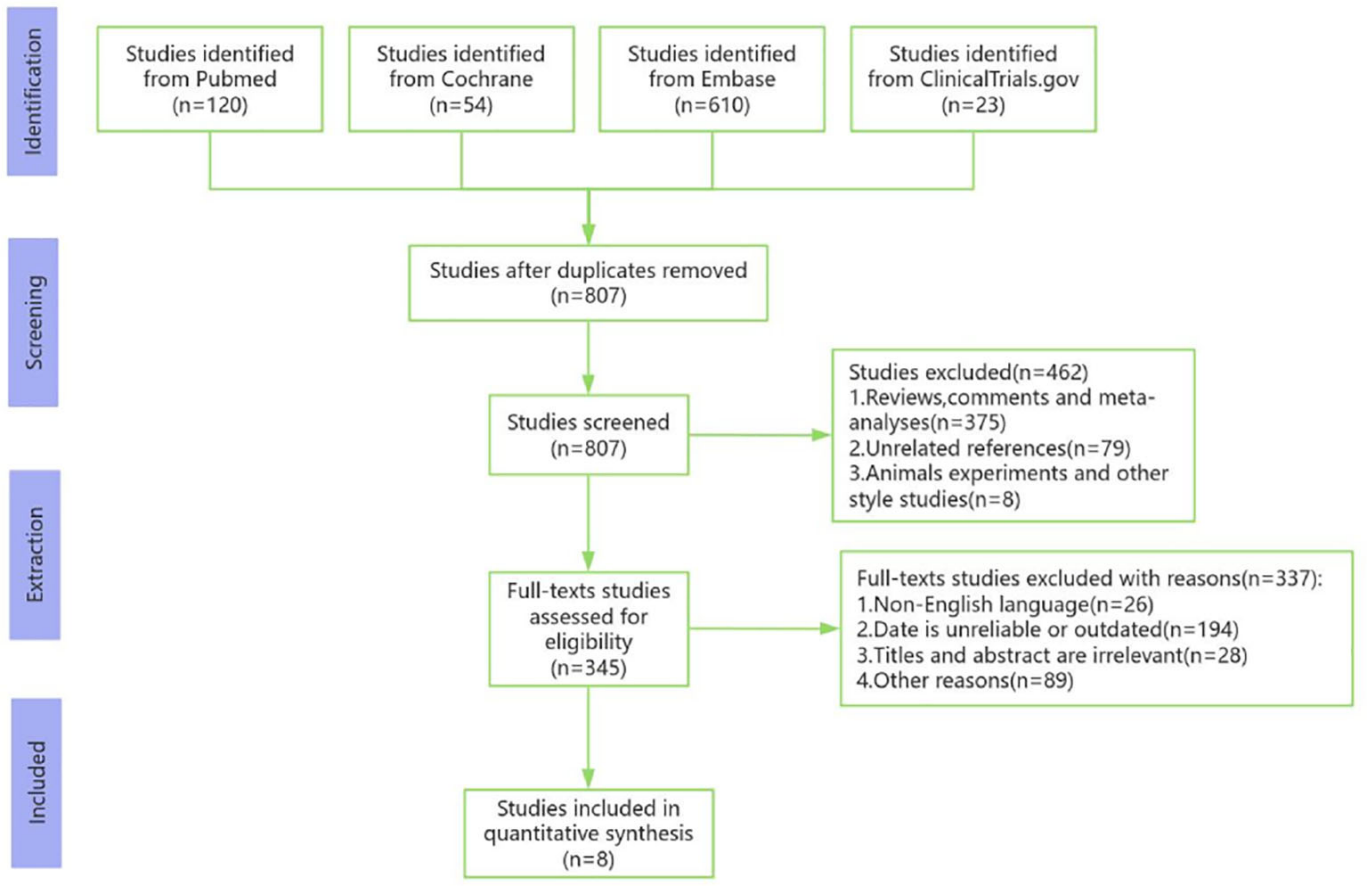
Flowchart of the literature search and specific screening process.

A total of 776 studies were initially retrieved from the databases. After removing the duplicates, titles, abstracts and full-text articles were carefully examined. Ultimately, 8 studies involving 306 participants were included in the meta-analysis (14–21). The flowchart of the literature search and screening procedure is shown in **Figure 1**, and specific details of the included studies are summarized in **Table 1**.

**Table 1:** Characteristics of studies incorporated in the meta-analysis.

Study	Study type	Sample size	Gender, male/female	Mean age, years	Median follow-up, months	Mean number of IP	Intervention (mg)	EGFR	Endpoints
Fan et al. (21)	Single-arm	132	42/90	52 (31-74 )	18	6	50	132	ORR、DCR、 OS、AEs
Fan et al. (17)	Single-arm	26	NR	54 (31-70)	11	4 (2-14)	50	26	ORR、DCR、OS、AEs
Geng et al. (18)	Prospective	34	11/23	54 (26-72)	3.5	3 (1-12)	15、20、25、30、40	27	ORR、DCR、OS、AEs
Li et al. (19)	Single-arm	23	10/13	54 (36-68)	NR	8 (2-16)	30、40、50	18	ORR、DCR、PFS、OS、AEs
Miao et al. (15)	Prospective	23	10/13	53 (38-74)	NR	4 (1-10)	10	16	ORR、DCR、PFS、OS、AEs
Pan et al. (14)	Single-arm	13	4/9	55 (37-71)	>4	6 (2-8)	10、15	10	ORR、DCR、NPFS、OS、AEs
Pan et al. (16)	Single-arm	21	NR	56 (43-73)	0.3-16.6	5	10	NA	ORR、DCR、mPFS、OS、AEs、
Zhou et al. (20)	Prospective	34	14/20	56 (37-73)	7.5	7 (2-22)	10、15、20、30、40、50	27	ORR、DCR、NPFS、OS、AEs

IP, Intrathecal pemetrexed; EGFR, Epidermal growth factor receptor; ORR, Objective response rate; DCR, Disease control rate; OS, overall survival; AEs, adverse events; PFS, progression-free survival; NPFS, Neurological progression-free survival.

### 3.2 Quality assessment

**Figure 2:**
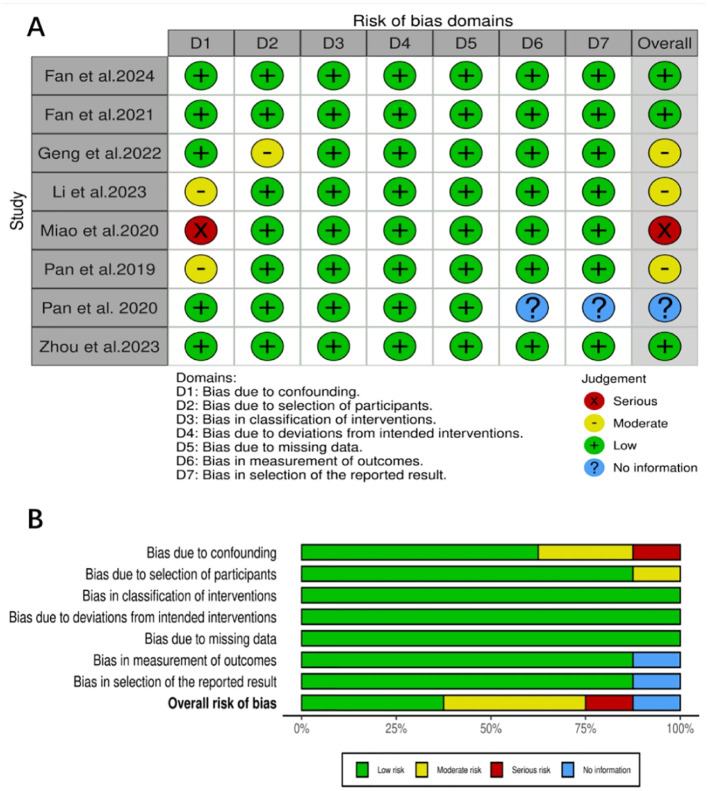
**(A)** Traffic plot of risk of bias, **(B)** Summary plot of risk of bias.

The eight studies (14–21) included in this research were all assessed using the ROBINS-I tool. Three studies had a low risk of bias, three had a moderate risk, one had a serious risk, and one was unclear. Detailed results of the quality assessment are shown in **Figures 2A, B**.

### 3.3 Meta-analysis results

**Figure 3:**
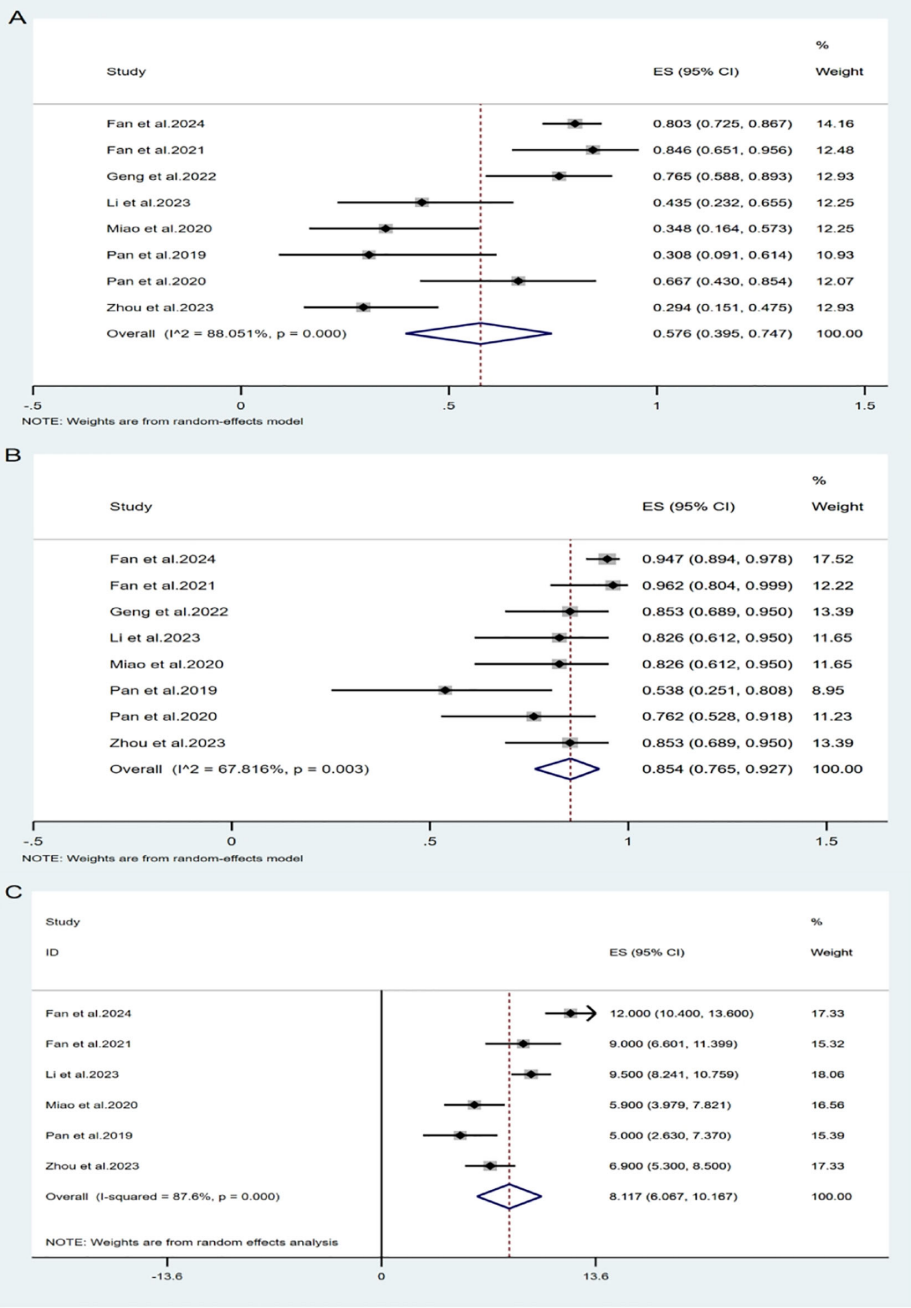
Pooled analysis of overall efficacy: **(A)** ORR, **(B)** DCR, **(C)** mOS.

All included studies (14–21) evaluated the efficacy of intrathecal pemetrexed in patients with NSCLC-LM. The ORR reported across studies ranged from 29.4% to 84.6%. Owing to significant study heterogeneity (I²= 88.05%, P= 0.000), so meta-analysis was conducted using a random effects model. The pooled ORR was 57.6% (95% CI: 39.5%-74.7%) (**Figure 3A**), indicating that pemetrexed demonstrates considerable efficacy in treating leptomeningeal metastasis from NSCLC, with an ORR of 57.6%.

All studies reported available data on DCR, which ranged from 53.8% to 96.2%. The pooled DCR was 85.4% (95% CI: 76.5%-92.7%) (**Figure 3B**), with notable heterogeneity (I² = 67.82%, P = 0.003).

Two studies did not report OS for all patients after administration of pemetrexed, only reporting the median OS (mOS) without the standard deviation. Therefore, only six studies (14, 15, 17, 19–21) were included in the OS analysis. Using a random-effects model (I² = 87.6%, P = 0.000), the pooled mOS was 8.12 months (95% CI: 6.07-10.17 months).

Regarding progression-free survival(PFS), three of the included studies did not report PFS, while two reported median neurological progression-free survival (NPFS), and one only reported the median PFS (mPFS). Due to the limited and inconsistent data on PFS, a meta-analysis of this endpoint was not feasible.

### 3.4 Subgroup analysis

Due to the heterogeneity observed in the overall meta-analysis, we further investigated two potential causes: drug dosage and genetic differences. Through subgroup analysis (**Supplementary Figure S5**), we found that while overall heterogeneity still persisted, the results became more stable, and the heterogeneity within some subgroups was reduced. The specific details are presented in **Table 2**.

**Table 2:** Subgroup analysis of the efficacy of pemetrexed in patients with NSCLC-LM.

Efficacy endpoints	Grouping basis	Group	ES,% (95% CI)	I^2^,100%	P	Total of I^2^/%	Total of P
ORR	Dosage	40-50mg	84.5 (70.0-95.6)	27.43	0.239	83.37	< 0.001
		10-30mg	46.6 (29.2-64.4)	76.49	< 0.001		
	Genetic differences	EGFR	56.2 (34.7-76.6)	89.04	< 0.001	82.56	< 0.001
		Other types	44.8 (25.1-65.1)	0.00	0.639		
DCR	Dosage	40-50mg	100 (98.5-100.0)	0.00	0.997	57.43	0.009
		10-30m	79.7 (72.2-86.4)	0.78	0.441		
	Genetic differences	EGFR	85.1 (73.3-94.2)	73.79	0.001	61.37	0.003
		Other types	76.6 (57.1-92.3)	0.00	0.960		

ES, Effect size.

### 3.5 Safety

We analyzed the most common AEs, both overall and those classified as Grade ≥ III, related to intrathecal chemotherapy using pemetrexed for NSCLC-LM (**Table 3**). All grades of AEs are shown in **Supplementary Figure S10**, while AEs of Grade ≥ III are shown in **Supplementary Figure S15**. Most patients reported Grade 1–2 AEs, indicating good tolerability among the majority. Adverse event data were unavailable for one study, resulting in the inclusion of seven studies (14, 15, 17–21). The analysis identified the three most common AEs: myelosuppression (32.6%, 95% CI: 24.0%-41.8%), headache (24.8%, 95% CI: 4.6%-53.0%), and abnormal transaminase levels (11.8%, 95% CI: 3.0%-24.4%). It was observed that two studies employed different treatment regimens to mitigate drug-related AEs: one study used leucovorin calcium in addition to the routine vitamins B12 and folate, and dexamethasone, while the other study used only dexamethasone. The remaining five studies all used the routine vitamins B12 and folate, and dexamethasone. The study including leucovorin calcium reported a significantly lower incidence of myelosuppression compared to the other six studies (11.8% vs. 34.3%). Further details are shown in the **Supplementary Figure S20**.

**Table 3:** AEs of the studies included in the meta-analysis.

AE	All grade ES,% (95CI)	I^2^,100%	Grade≥III ES,% (95CI)	I^2^,100%
Myelosuppression	32.6 (24.0-41.8)	50.93	17.4 (9.7-26.4)	54.56
Nausea	7.3 (3.4-12.1)	19.62	NA	NA
Vomiting	11.7 (5.7-19.2)	47.18	NA	NA
ALT/AST	11.8 (3.0-24.4)	80.99	1.7 (0.0-8.4)	45.95
Neurotoxicity				
Radiculitis	8.4 (1.5-18.8)	69.28	3.5 (0.0-14.7)	68.61
Headache	24.8 (4.6-53.0)	94.90	3.6 (0.0-11.0)	61.88
Leukoencephalopathy	6.4 (2.9-10.9)	0.00	NA	NA

NA, Not assessable; ALT, Alanine aminotransferase; AST, Aspartate aminotransferase.

### 3.6 Sensitivity analysis

To assess the stability of the meta-analyses results and determine whether any individual study influenced the overall results, we performed sensitivity analyses. The results of the analyses showed only minor changes in the overall findings regardless of which studies were excluded, indicating that our results were reliable. Further details can be found in the **Supplementary Material**.

### 3.7 Publication bias

Egger’s and Begg’s tests were used to evaluate publication bias in order to confirm the stability of the meta-analysis results. The results were generally consistent with the overall findings, however, in the case of myelosuppression (Grade ≥ III), evidence of publication bias was observed (Egger’s test: t=4.68, P=0.009 < 0.05). The trim and filling method was utilized to evaluate stability. Heterogeneity testing revealed Q=1.555, P=0.907, indicating the use of a fixed-effects model with a logOR =0.148, 95% CI: 0.025-0.271. After four iterations using the Linear method, the software estimated three missing counts. Including data from the 3 dummy studies, a meta-analysis was performed on all the studies, revealing heterogeneity (Q = 3.607, P = 0.891) under the fixed-effects model, with logOR = 1.118, 95% CI: 1.001-1.248. The results were still statistically significant after three additional investigations were included, suggesting that they were comparatively stable. The publication bias for myelosuppression (Grade ≥ III) may originate from researchers’ selective reporting of significant results and the occurrence of chance extreme data in small-sample studies. For DCR, evidence of publication bias was also found (Egger’s test: t= -3.23, P= 0.018 < 0.05). To identify the cause of publication bias, subgroup analyses were performed, and subsequent Egger’s test yielded t= -1.11, P= 0.296 > 0.05, suggesting that the publication bias of DCR was most likely related to drug dosage. Please refer to the **Supplementary Material** for more details.

## 4 Discussion

EGFR mutations in NSCLC primarily occur in females, non-smokers, patients with adenocarcinoma, and Japanese patients (22). The main treatment methods for NSCLC-LM patients include radiotherapy (RT), systemic and intrathecal chemotherapy, molecular targeted therapies and first-generation EGFR tyrosine kinase inhibitors (TKIs). Despite these interventions, prognosis remains poor. A study involving 184 NSCLC patients with secondary LM showed that those receiving TKI treatment possessed a longer OS than those without. Interestingly, 42 individuals who received whole brain radiation therapy (WBRT) did not have longer OS than those who did not, and survival was not improved by WBRT plus TKI (23). In a single-center retrospective analysis of 136 LM patients with EGFR-mutated NSCLC, TKI therapy was again associated with longer OS, while WBRT did not confer additional OS benefits (24). Osimertinib, a third-generation EGFR-TKI, demonstrates strong penetration of the blood-brain barrier and is a treatment for NSCLC with EGFR mutations. A study of 27 NSCLC patients with LM treated with osimertinib showed an ORR of 55%-62%, a mPFS of 8–11 months (25). Immune checkpoint inhibitor (ICIs) have significantly altered the management landscape for NSCLC. A prospective study demonstrated that the median PFS for 19 NSCLC patients with LM treated with ICIs was 3.7 months, with 6-month and 12-month OS rates of 36.8% and 21.1%, respectively (26). In asymptomatic, untreated patients with brain metastases, bevacizumab, a humanized monoclonal antibody that targets vascular endothelial growth factor (VEGF), has demonstrated effectiveness when combined with paclitaxel and carboplatin, with a response rate as high as 61.2% (27).

Commonly used intrathecal chemotherapy agents include MTX, Ara-C, and thiotepa. According to a pooled analysis, patients who receive only IC have a clinical response rate of 71%-79% and a median survival time of 7.5 months, which is noticeably longer than that of patients receiving multiple interventions. This suggests that intrathecal chemotherapy is an effective treatment for NSCLC-LM (28). Although MTX is the most studied drug for LM treatment, optimal administration times and treatment durations remain undetermined. Compared to combination therapy, MTX monotherapy can prolong the survival of LM patients, but adverse reactions such as leukoencephalopathy may occur (29). Therefore, when used in combination therapy, MTX is administered 2 to 3 weeks prior to WBRT. A randomized study involving 28 patients showed that liposome-coated Ara-C resulted in improved mPFS and OS (30).

Pemetrexed is a multi-target anti-folate metabolic chemotherapy drug that plays an important function in the treatment of LM from NSCLC. This meta-analysis included eight clinical studies, encompassing 306 patients to assess the safety and efficacy of intrathecal pemetrexed in NSCLC-LM treatment. The pooled analysis showed promising efficacy, with an ORR of 57.6%, a DCR of 85.4%, and a mOS of 8.12 months, despite variations in patient conditions, treatment modalities, and the number of intrathecal chemotherapies among the studies. Further subgroup analysis revealed that pemetrexed at a dosage of 40–50 mg demonstrated superior efficacy, with an ORR of 84.5% (95% CI: 70%-95.6%). For patients with EGFR mutations, the ORR was 56.2% (95% CI: 34.7%-76.6%). Pemetrexed not only demonstrated identified efficacy in the treatment of NSCLC-LM but also possesses a reasonable safety profile. Our study identified the most common AEs as bone marrow suppression (32.6%), headache (24.8%), and elevated liver enzymes (11.8%). The majority of AEs were classified as Grade 1-2, with occurrence of Grade ≥ III rarely exceeded 10%. Consequently, the safety of pemetrexed in NSCLC-LM treatment appears acceptable.

This study has several limitations. Firstly, the included studies are small-sample, non-controlled trials with low levels of evidence, limiting our ability to draw definitive conclusions regarding efficacy and adverse events. Secondly, high heterogeneity among studies exists. Although subgroup analyses were conducted based on drug dosage and gene mutation status, other factors such as the number of intrathecal treatments, baseline characteristics, gender, and age may also contribute to the heterogeneity. Lastly, reporting of smoking history, baseline lung function, and performance status was not inconsistent among the included studies, affecting the analysis of these elements. Furthermore, all of the research was conducted in China, which would restrict how broadly our results can be applied to non-Asian NSCLC-LM patients. Large-scale RCTs are necessary to confirm pemetrexed’s therapeutic efficacy in the intended population.

In conclusion, our meta-analysis indicates the efficacy and safety of intrathecal pemetrexed chemotherapy for patients with leptomeningeal metastasis from non-small cell lung cancer, providing a groundwork for its clinical application. Nevertheless, due to the restricted clinical data, further large-scale RCTs are necessary to validate these findings.

## Data Availability

The original contributions presented in the study are included in the article/**Supplementary Material**. Further inquiries can be directed to the corresponding authors.
