# Correction: The efficacy and safety of intrathecal pemetrexed for leptomeningeal metastasis from non-small cell lung cancer: a single-arm meta-analysis of Chinese patients

**DOI:** 10.3389/fonc.2025.1660077

**Published:** 2025-07-23

**Authors:** Yushi Zhao, Xueqin Gao, Yong Han

**Affiliations:** Department of Pharmacy, Union Hospital, Tongji Medical College, Huazhong University of Science and Technology, Wuhan, China

**Keywords:** pemetrexed, non-small cell lung cancer, leptomeningeal metastasis, meta-analysis, efficacy, safety

In the published article, there was an error in Affiliation 1. Instead of “Institude of Advanced Pharmaceutical Technology, Academy of Nutrition and Health, Hubei Province Key Laboratory of Occupational Hazard Identification and Control, College of Medicine, Wuhan University of Science and Technology, Wuhan, China”, it should be “Department of Pharmacy, Union Hospital, Tongji Medical College, Huazhong University of Science and Technology, Wuhan, China”.

In the published article, there was an error in the author list and citation. “Ting Wan” was erroneously added to the author list and citation. The author list and citation have been updated.

In the published article, there was an error in correspondence. “Ting Wan” was erroneously listed as a corresponding author. The only corresponding author is “Yong Han, hanyong@hust.edu.cn”.

In the published article, there was an error in the **Author contributions**. The correct **Author contributions** appears below: “YZ: Data curation, Formal Analysis, Writing – original draft. XG: Data curation, Formal Analysis, Writing – original draft. YH: Conceptualization, Writing – review & editing.”

The authors apologize for these errors and state that these do not change the scientific conclusions of the article in any way. The original article has been updated.

