# Supplementary material for: The efficacy and safety of intrathecal pemetrexed for leptomeningeal metastasis from non-small cell lung cancer: a single-arm meta-analysis of Chinese patients
Source: Front Oncol. 2025 Jun 18;15:1543416. doi: 10.3389/fonc.2025.1543416 (PMC12213478; doi:10.3389/fonc.2025.1543416)
Supplement: Supplementary file 1 [file DataSheet1.zip › Supplementary_Material.docx]

Supplementary Material

# Supplementary Tables

Table S1. Search strategies

| Databases | Search strategies | Result |
| --- | --- | --- |
| PubMed | ((((Pemetrexed[Title/Abstract]) OR (Alimta[Title/Abstract])) OR (LY 231514[Title/Abstract])) OR (MTA[Title/Abstract])) AND (((((leptomeningeal metastases[Title/Abstract]) OR (leptomeningeal metastasis[Title/Abstract])) OR (carcinomatous meningitis[Title/Abstract])) OR (CNS[Title/Abstract])) OR (central nervous system[Title/Abstract])) | 118 |
| Cochrane library | #1 MeSH descriptor: [Pemetrexed] explode all trees  #2 (Alimta):ti,ab,kw OR (MTA):ti,ab,kw OR (LY 231514):ti,ab,kw 1254  #3 #1 or #2  #4 (leptomeningeal metastases):ti,ab,kw OR (leptomeningeal metastasis):ti,ab,kw OR (carcinomatous meningitis):ti,ab,kw OR (CNS):ti,ab,kw OR (central nervous system):ti,ab,kw  #5 #3 and #4 | 54 |
| EMBASE | #5. #3 AND #4  #4. 'leptomeningeal metastases':ab,ti OR  'leptomeningeal metastasis':ab,ti OR  'carcinomatous meningitis':ab,ti OR 'cns':ab,ti  OR 'central nervous system':ab,ti  #3. #1 OR #2  #2. 'alimta':ab,ti OR 'mta':ab,ti OR 'ly  231514':ab,ti  #1. 'pemetrexed'/exp | 583 |
| ClinicalTrials.gov | Studies found for: leptomeningeal metastases \|pemetrexed | 21 |

# Supplementary Figures


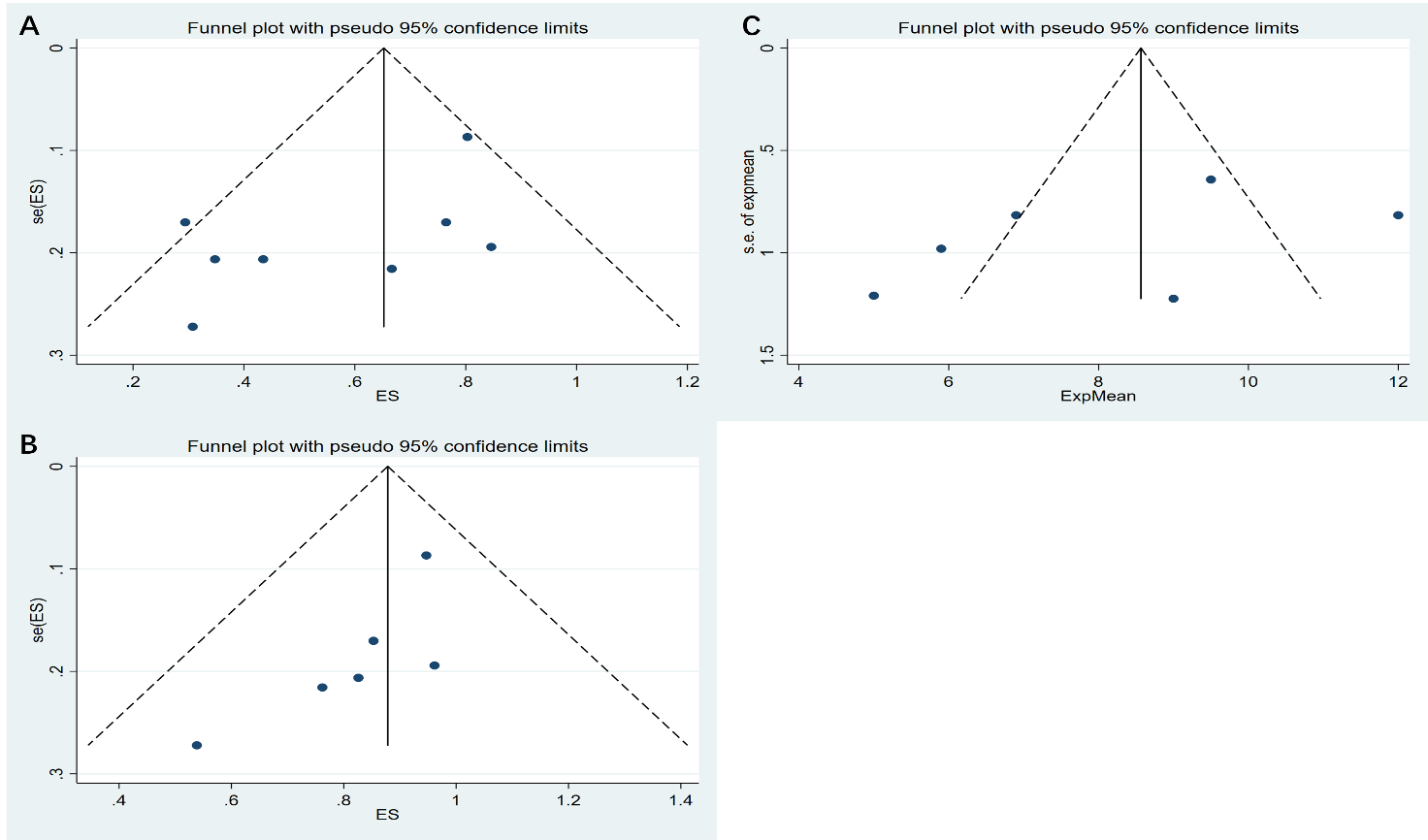


Figure S1. Funnel plots for different outcome indicators: (A) ORR, (B) DCR, (C) mOS


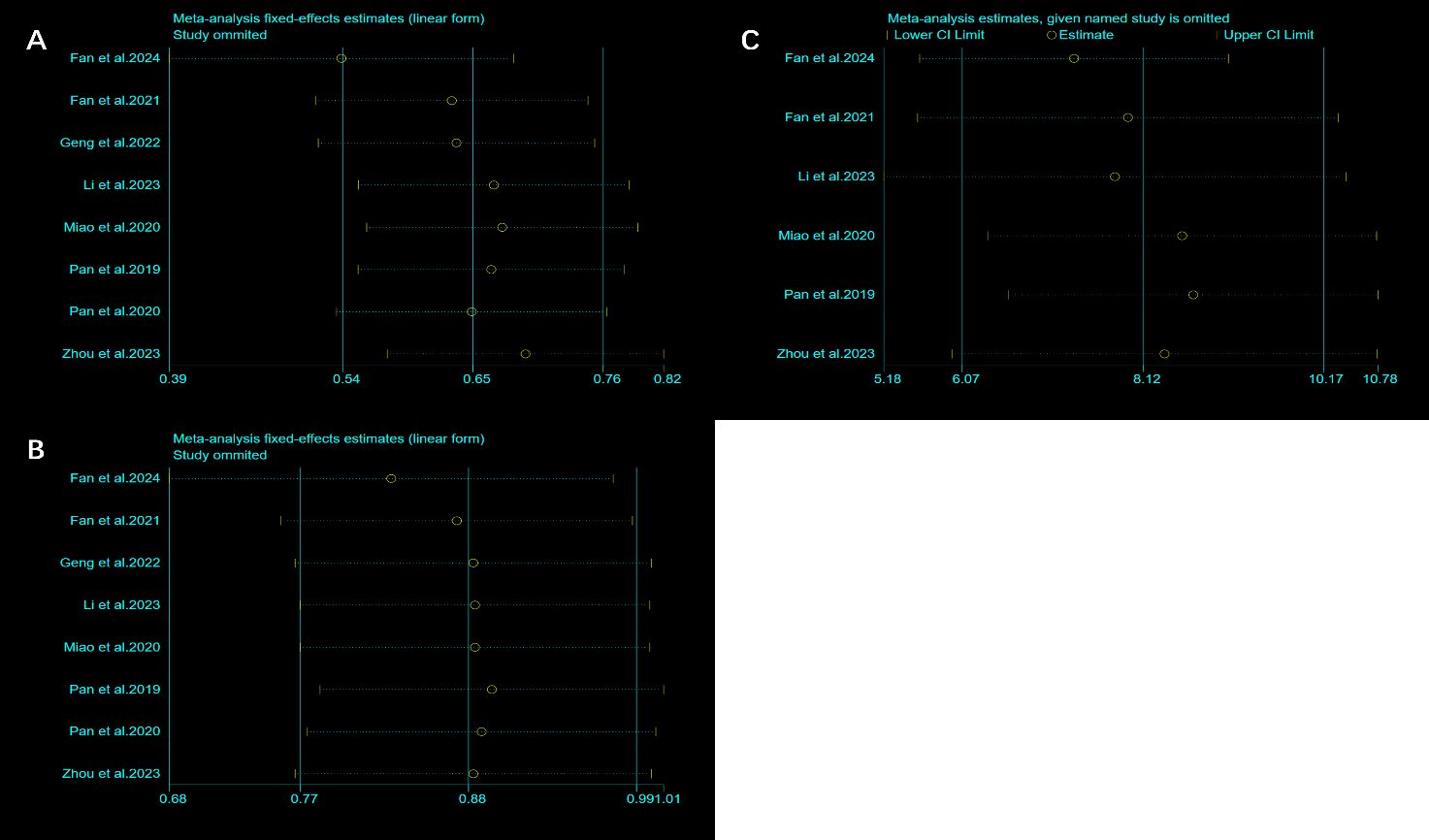


Figure S2. Sensitivity analysis of different outcome indicators: (A) ORR, (B) DCR, (C) mOS


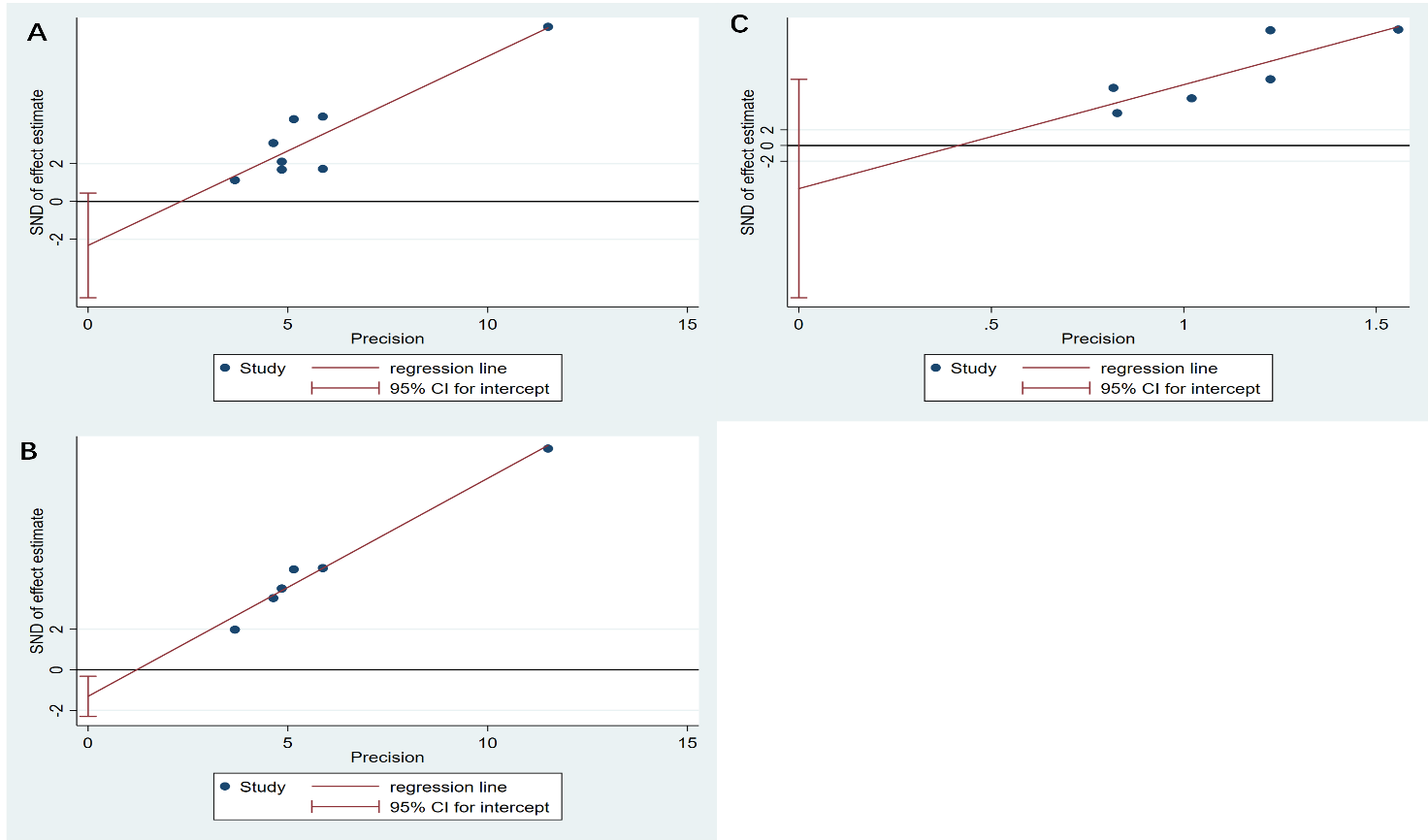


Figure S3. Egger's test for different outcome indicators: (A) ORR, (B) DCR, (C) mOS


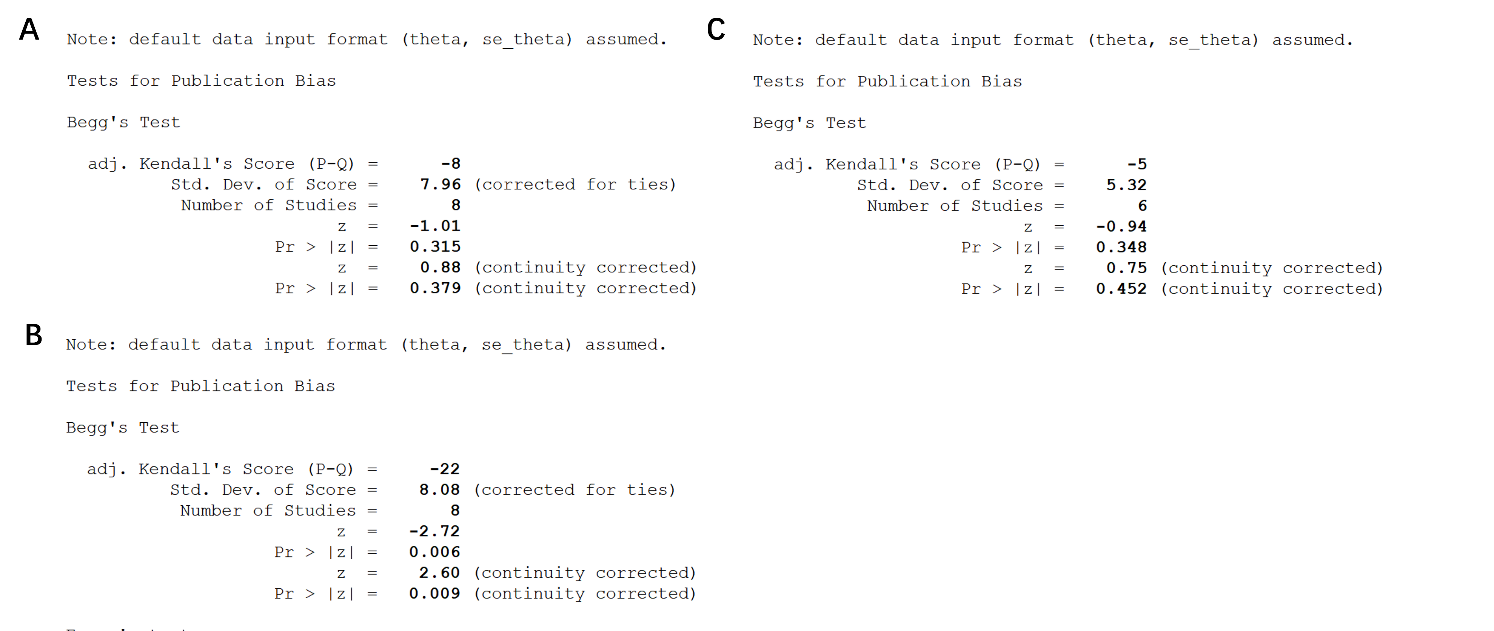


Figure S4. Begg's test for different outcome indicators: (A) ORR, (B) DCR, (C) mOS


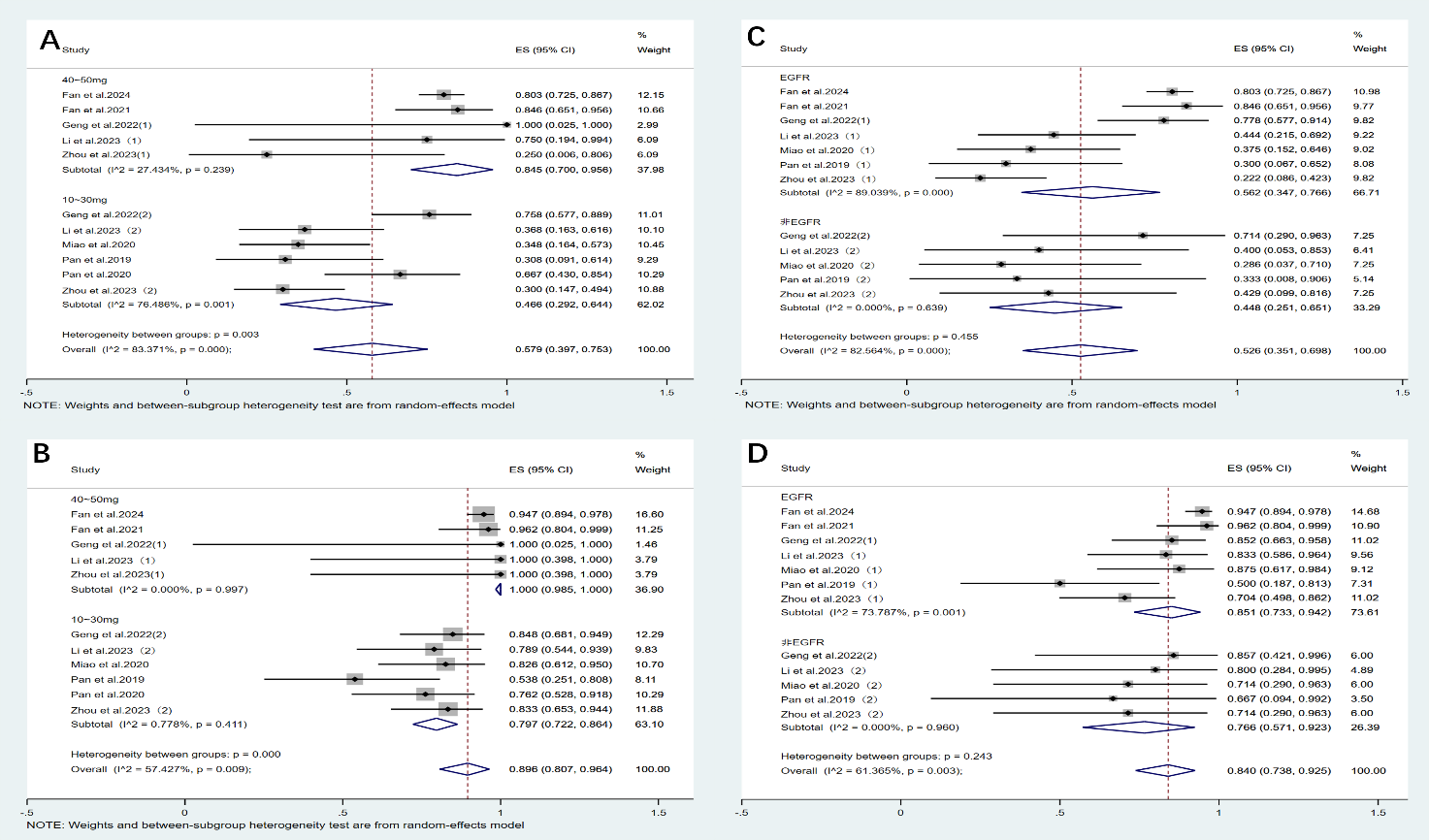


Figure S5. Forest plots for subgroup analyses: (A) dosage (ORR), (B) dosage (DCR), (C) Genetic differences (ORR), (D) Genetic differences (DCR)


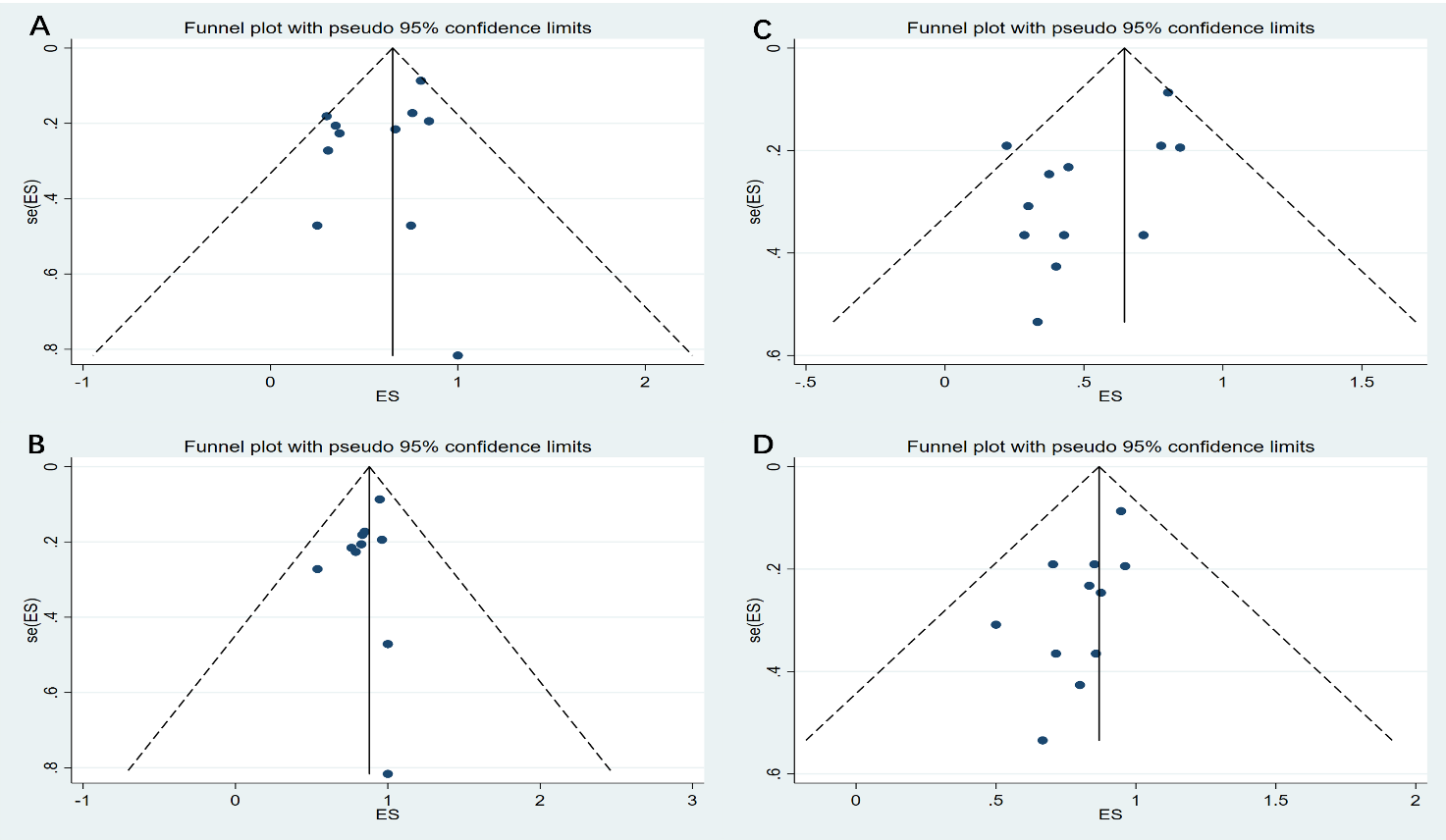


Figure S6. Funnel plots for subgroup analyses: (A) dosage (ORR), (B) dosage (DCR), (C) Genetic differences (ORR), (D) Genetic differences (DCR)


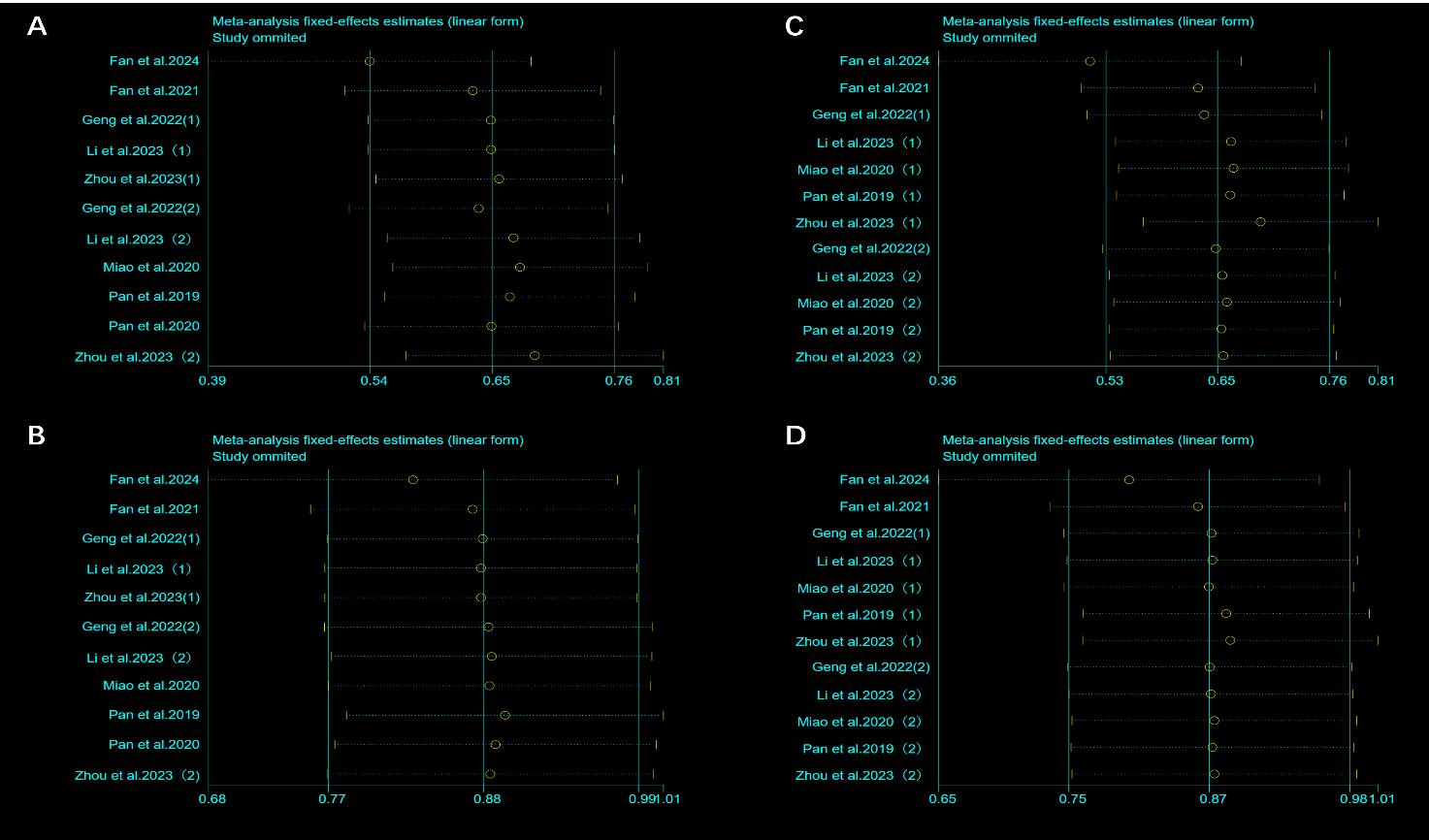


Figure S7. Sensitivity analyses for subgroup analyses: (A) dosage (ORR), (B) dosage (DCR), (C) Genetic differences (ORR), (D) Genetic differences (DCR)


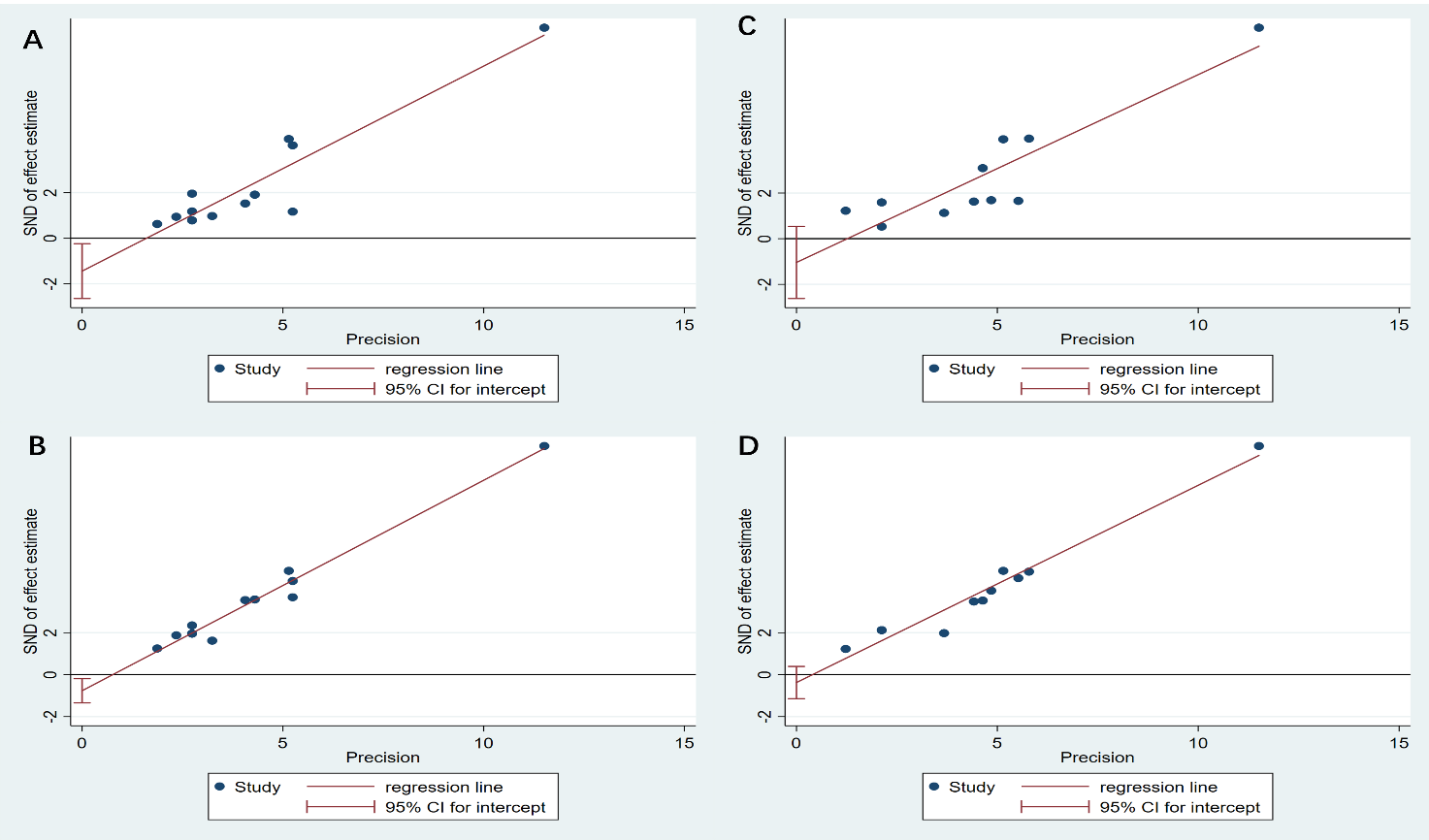


Figure S8. Egger's test for subgroup analysis: (A) dosage (ORR), (B) dosage (DCR), (C) Genetic differences (ORR), (D) Genetic differences (DCR)


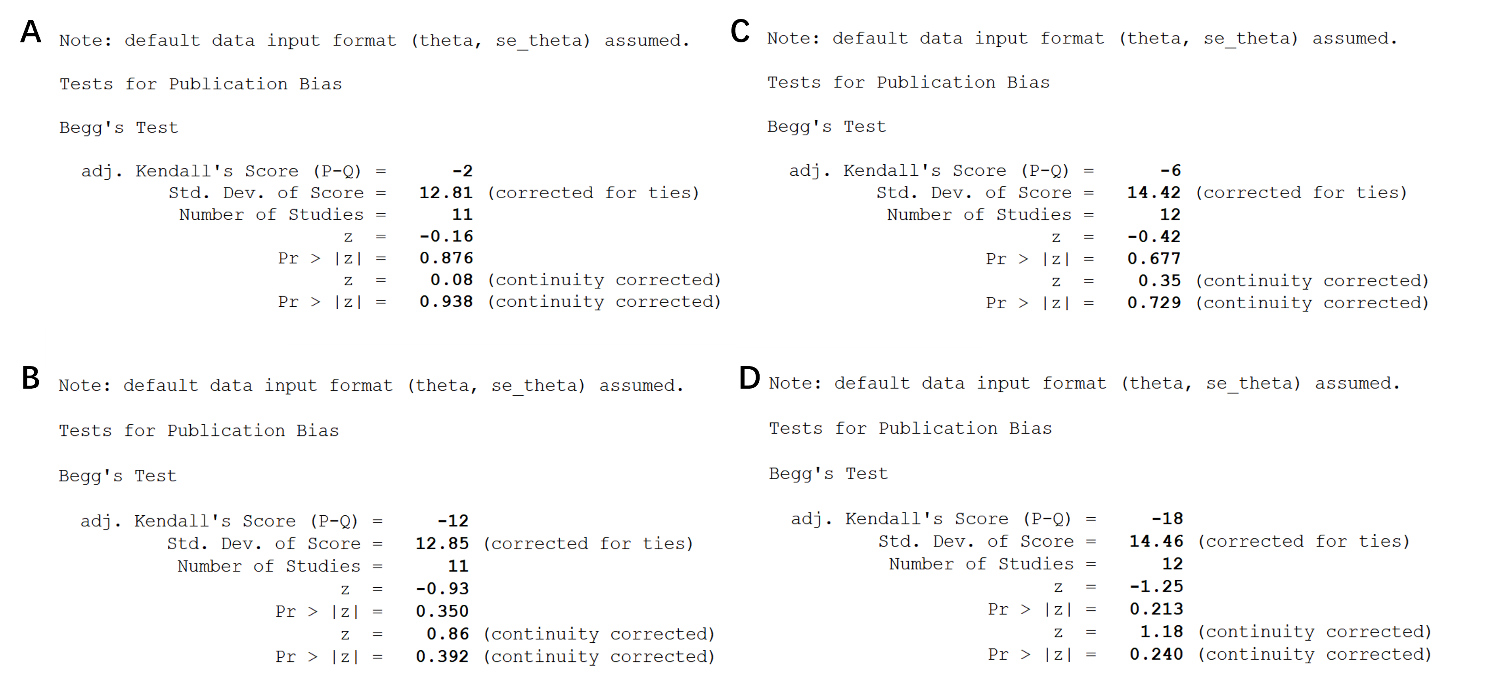


Figure S9. Begg's test for subgroup analysis: (A) dosage (ORR), (B) dosage (DCR), (C) Genetic differences (ORR), (D) Genetic differences (DCR)


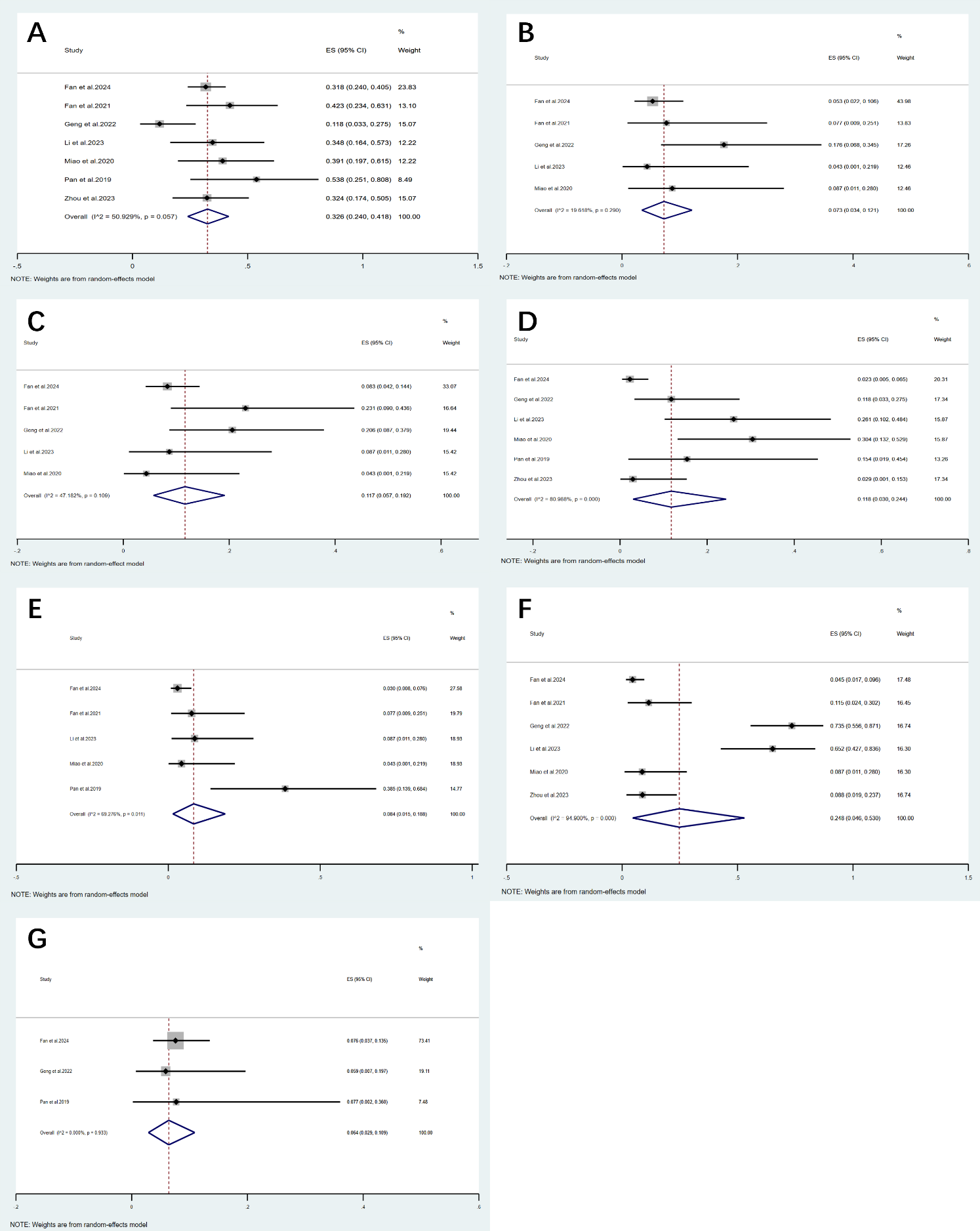


Figure S10. Forest plots of the most common AEs (All grades): (A) Myelosuppression, (B) Nausea, (C) Vomiting, (D) ALT/AST, (E) Radiculitis, (F) Headache, (G) Leukoencephalopathy


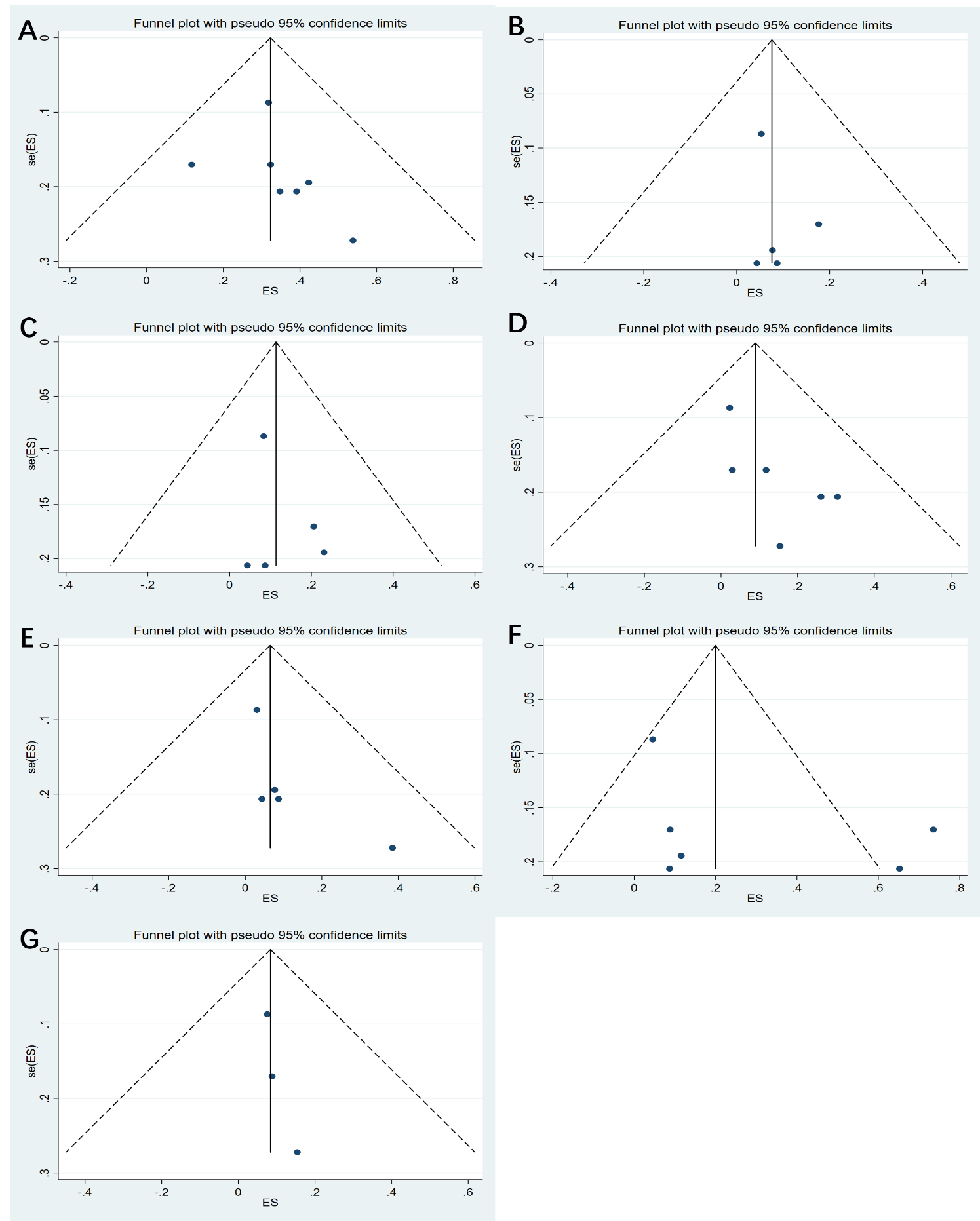


Figure S11. Funnel plots of the most common AEs (All grades): (A) Myelosuppression, (B) Nausea, (C) Vomiting, (D) ALT/AST, (E) Radiculitis, (F) Headache, (G) Leukoencephalopathy


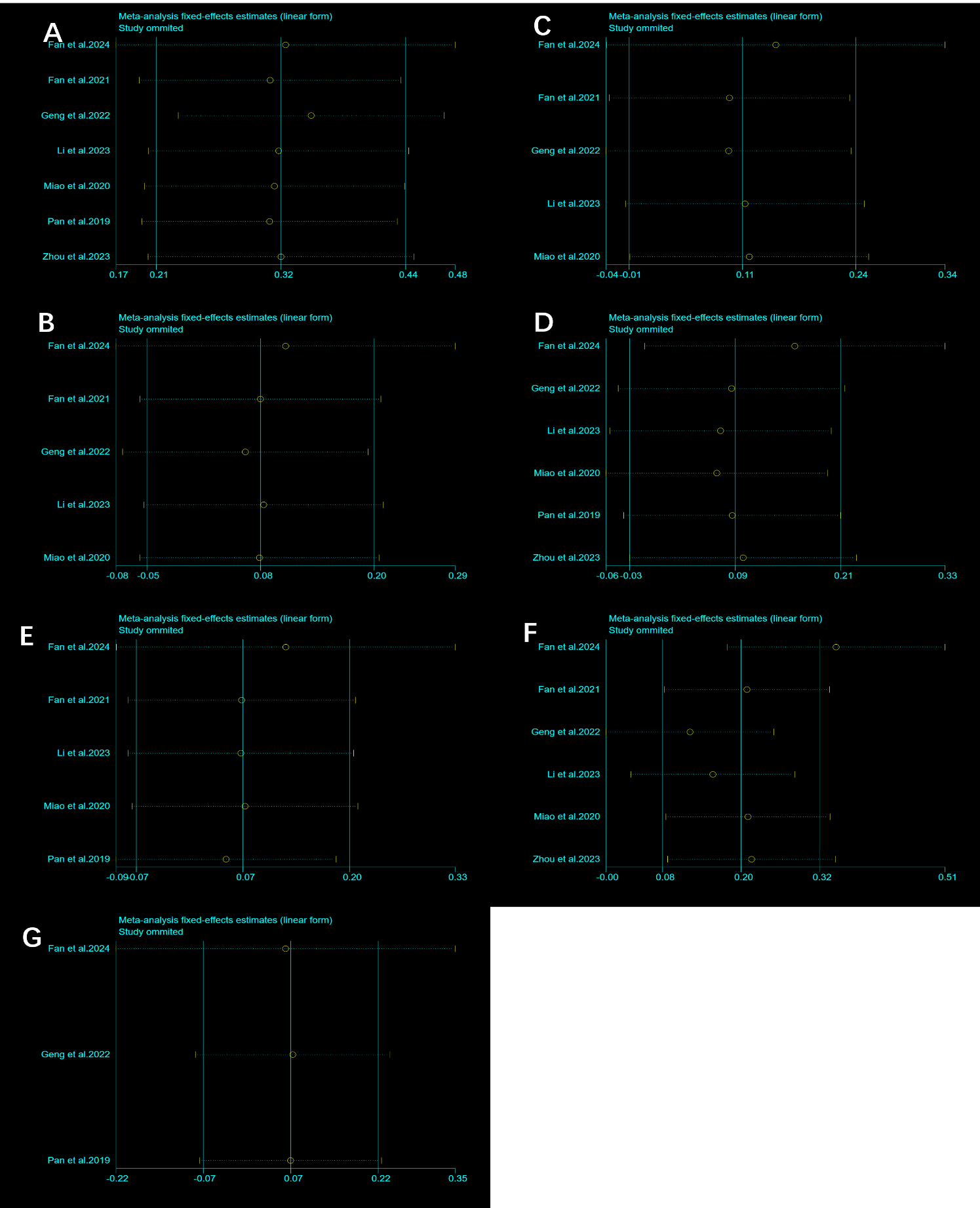


Figure S12. Sensitivity analyses for the most common AEs ( All grades): (A) Myelosuppression, (B) Nausea, (C) Vomiting, (D) ALT/AST, (E) Radiculitis, (F) Headache, (G) Leukoencephalopathy


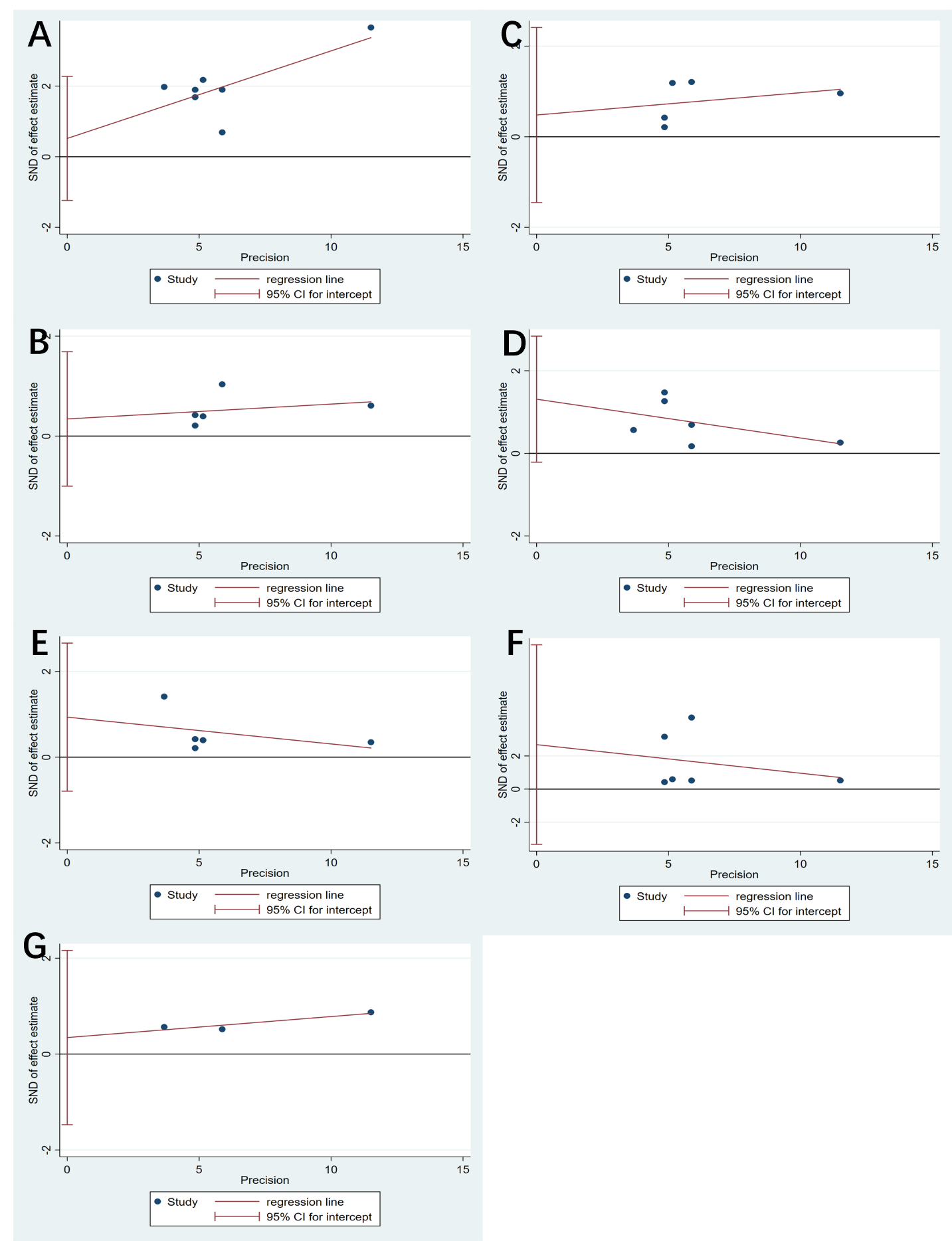


Figure S13. Egger's test for the most common AEs ( All grades): (A) Myelosuppression, (B) Nausea, (C) Vomiting, (D) ALT/AST, (E) Radiculitis, (F) Headache, (G) Leukoencephalopathy


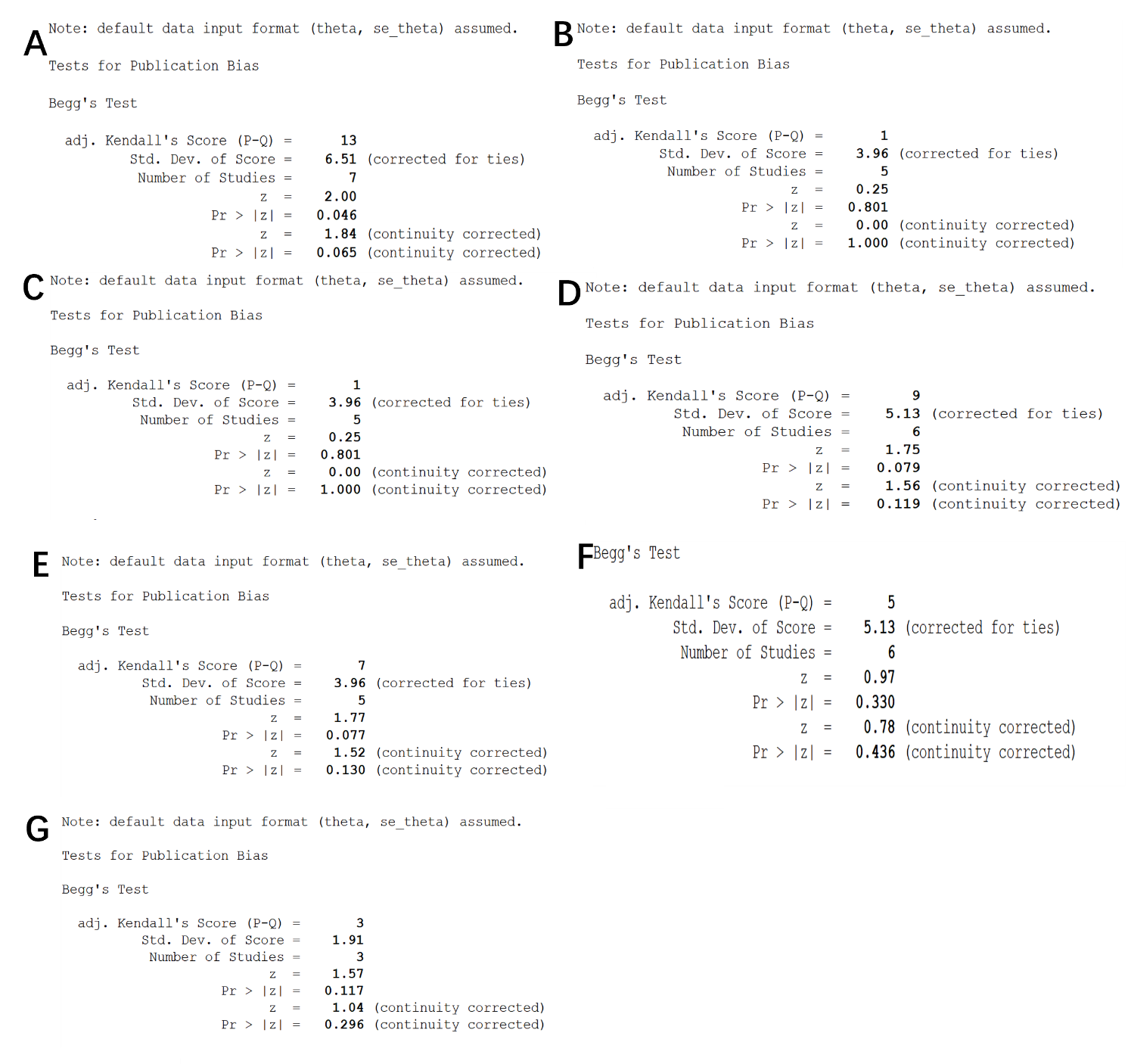


Figure S14. Begg's test for the most common AEs ( All grades): (A) Myelosuppression, (B) Nausea, (C) Vomiting, (D) ALT/AST, (E) Radiculitis, (F) Headache, (G) Leukoencephalopathy


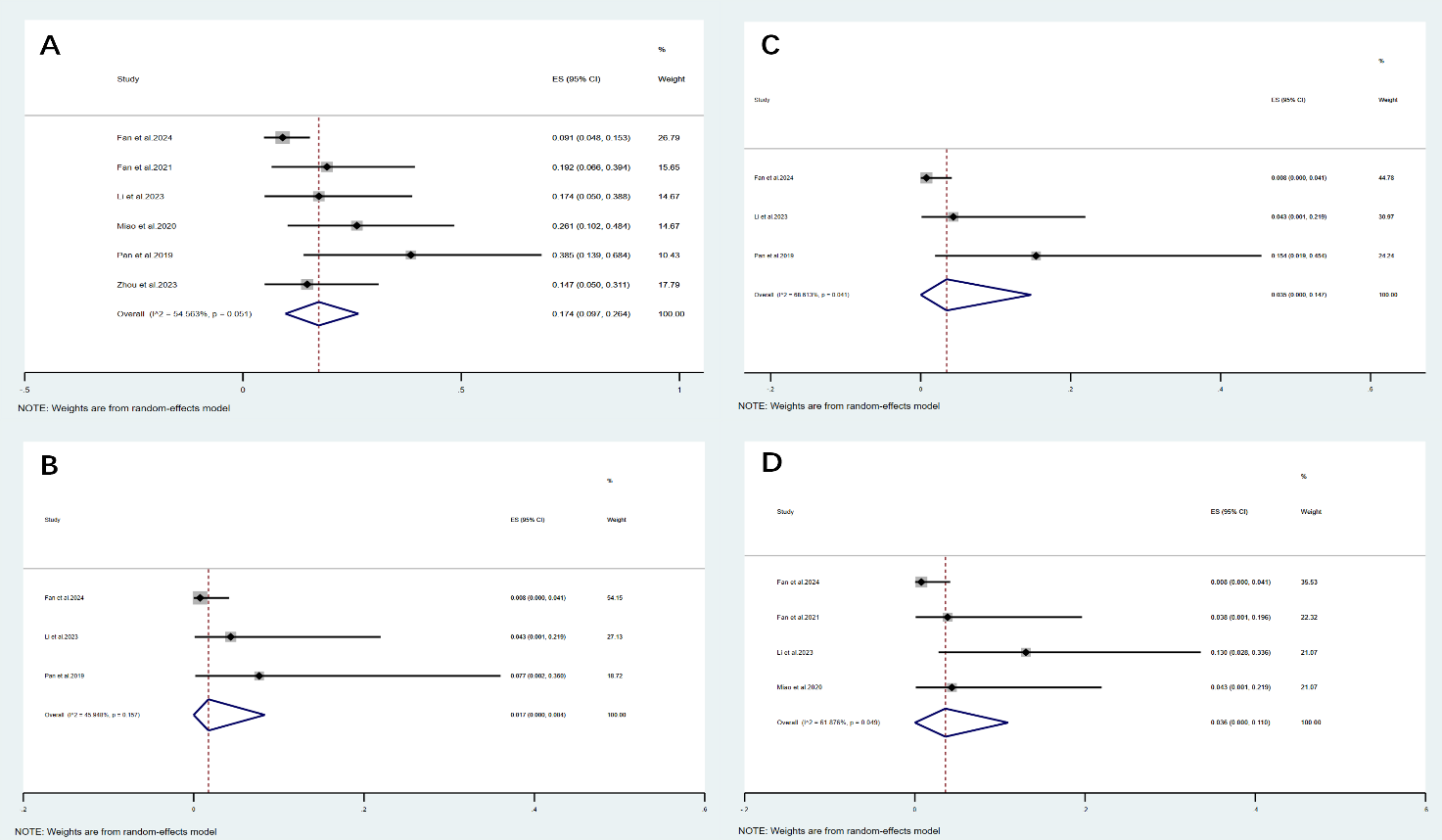


Figure S15. Forest plots of the most common AEs (Grade≥ III ): (A) Myelosuppression, (B) ALT/AST, (C) Radiculitis, (D) Headache


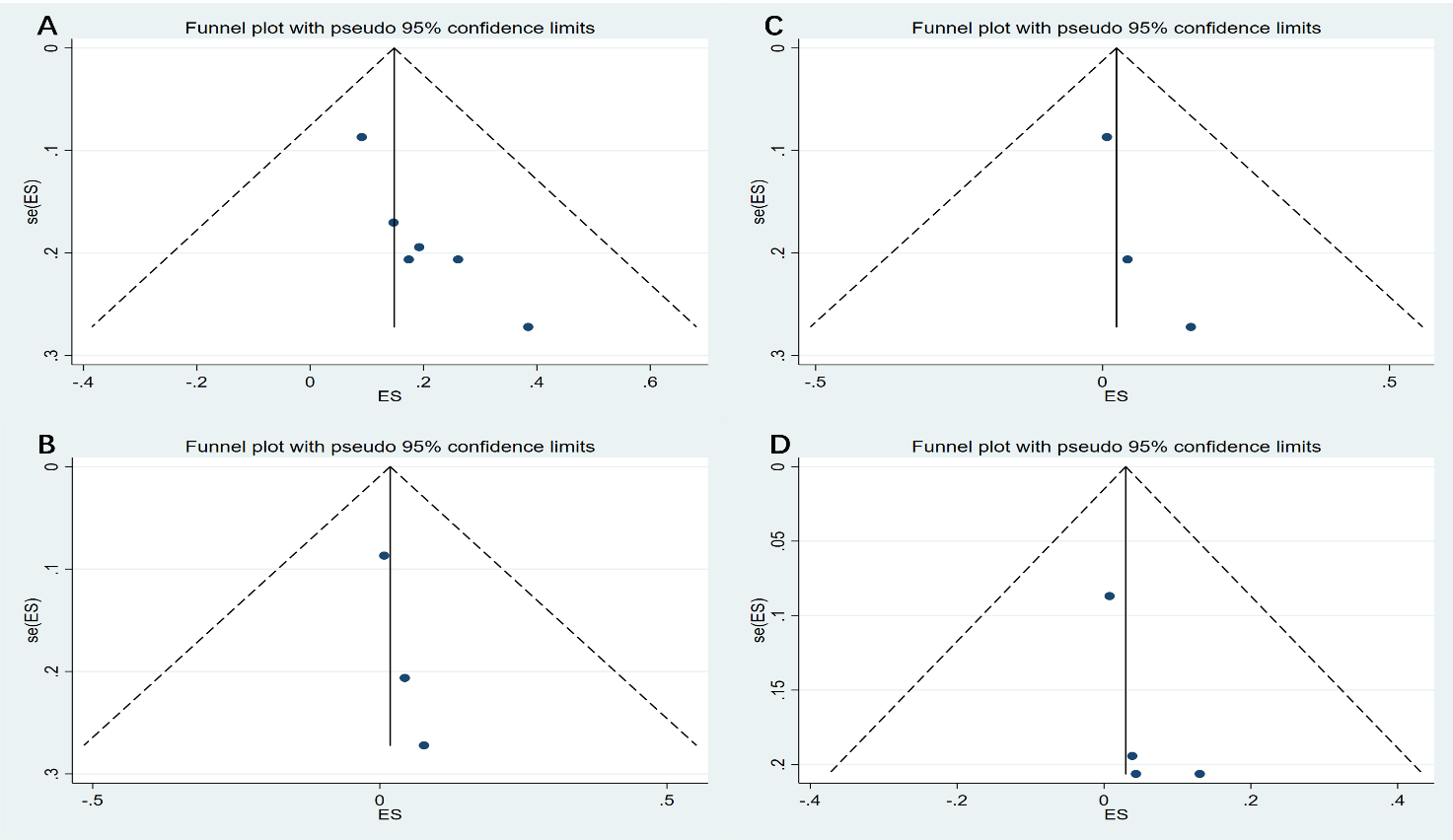


Figure S16. Funnel plots of the most common AEs (Grade≥ III ): (A) Myelosuppression, (B) ALT/AST, (C) Radiculitis, (D) Headache


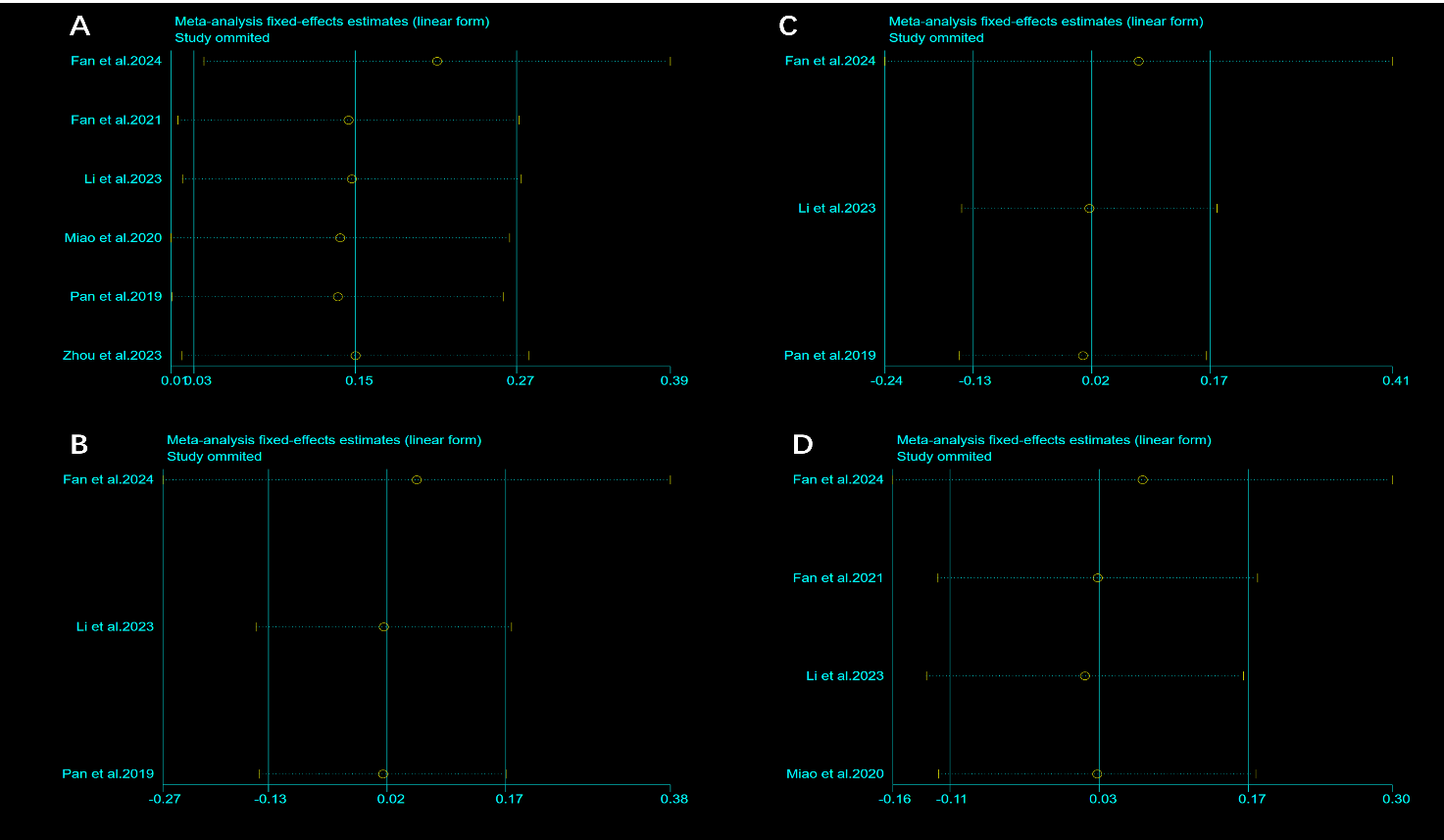


Figure S17. Sensitivity analyses for the most common AEs (Grade≥ III ): (A) Myelosuppression, (B) ALT/AST, (C) Radiculitis, (D) Headache


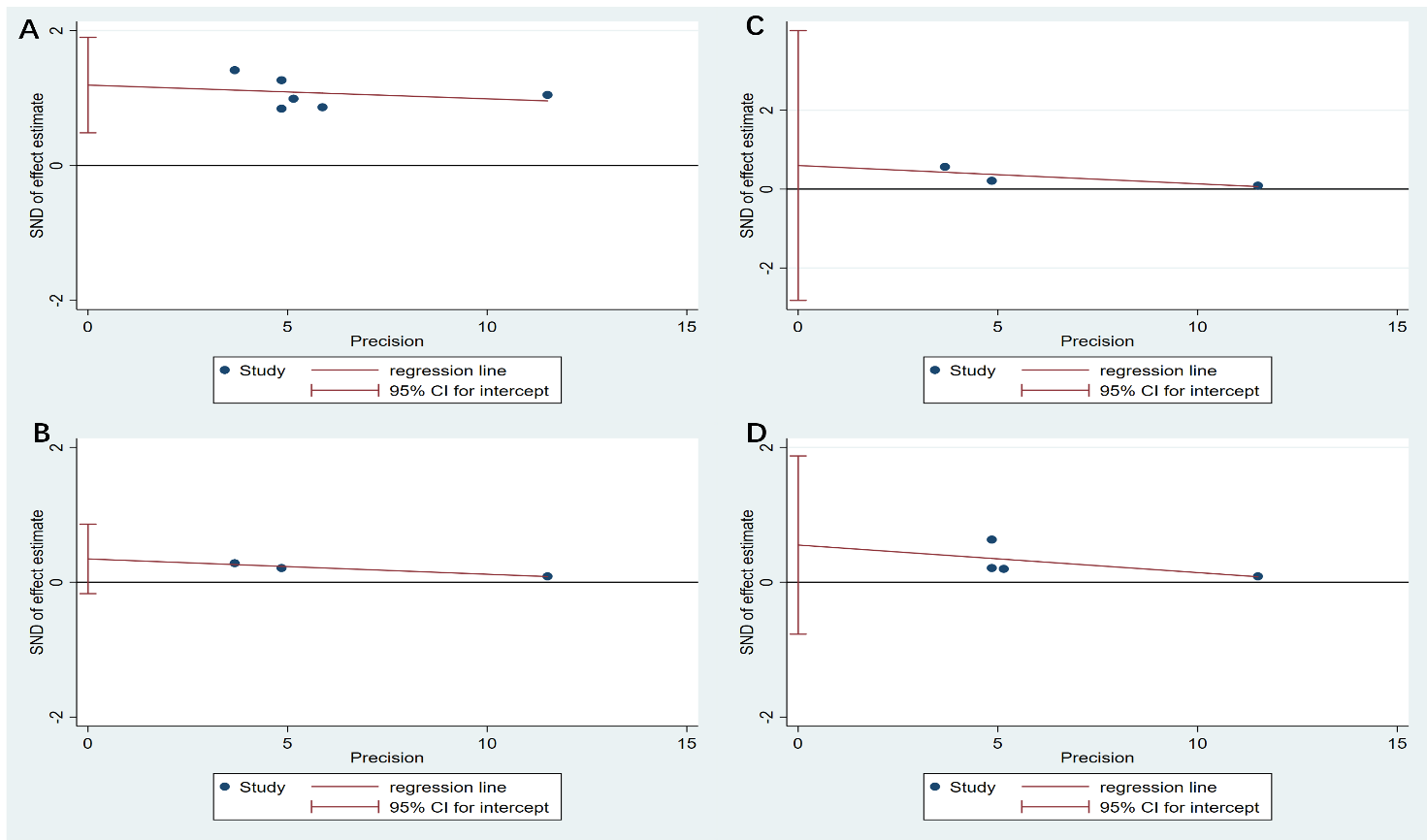


Figure S18. Egger's test for the most common AEs (Grade≥ III ): (A) Myelosuppression, (B) ALT/AST, (C) Radiculitis, (D) Headache


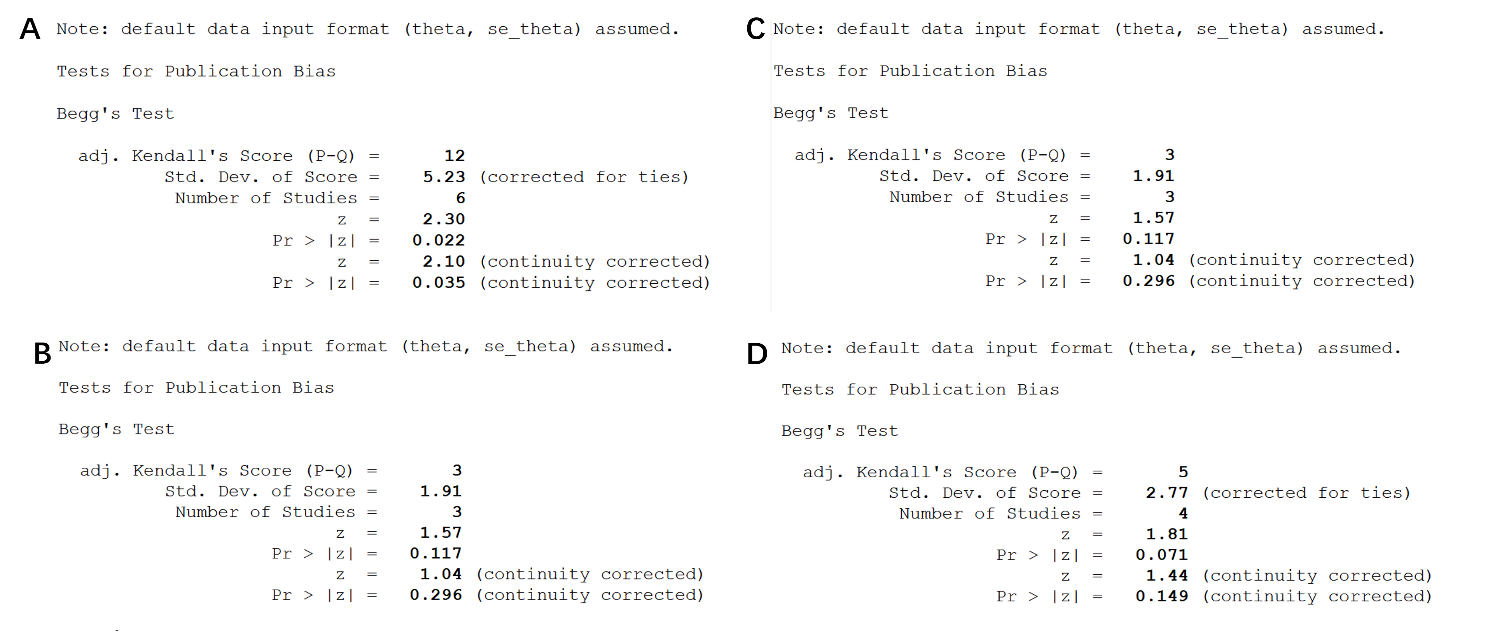


Figure S19. Begg's test for the most common AEs (Grade≥ III ): (A) Myelosuppression, (B) ALT/AST, (C) Radiculitis, (D) Headache


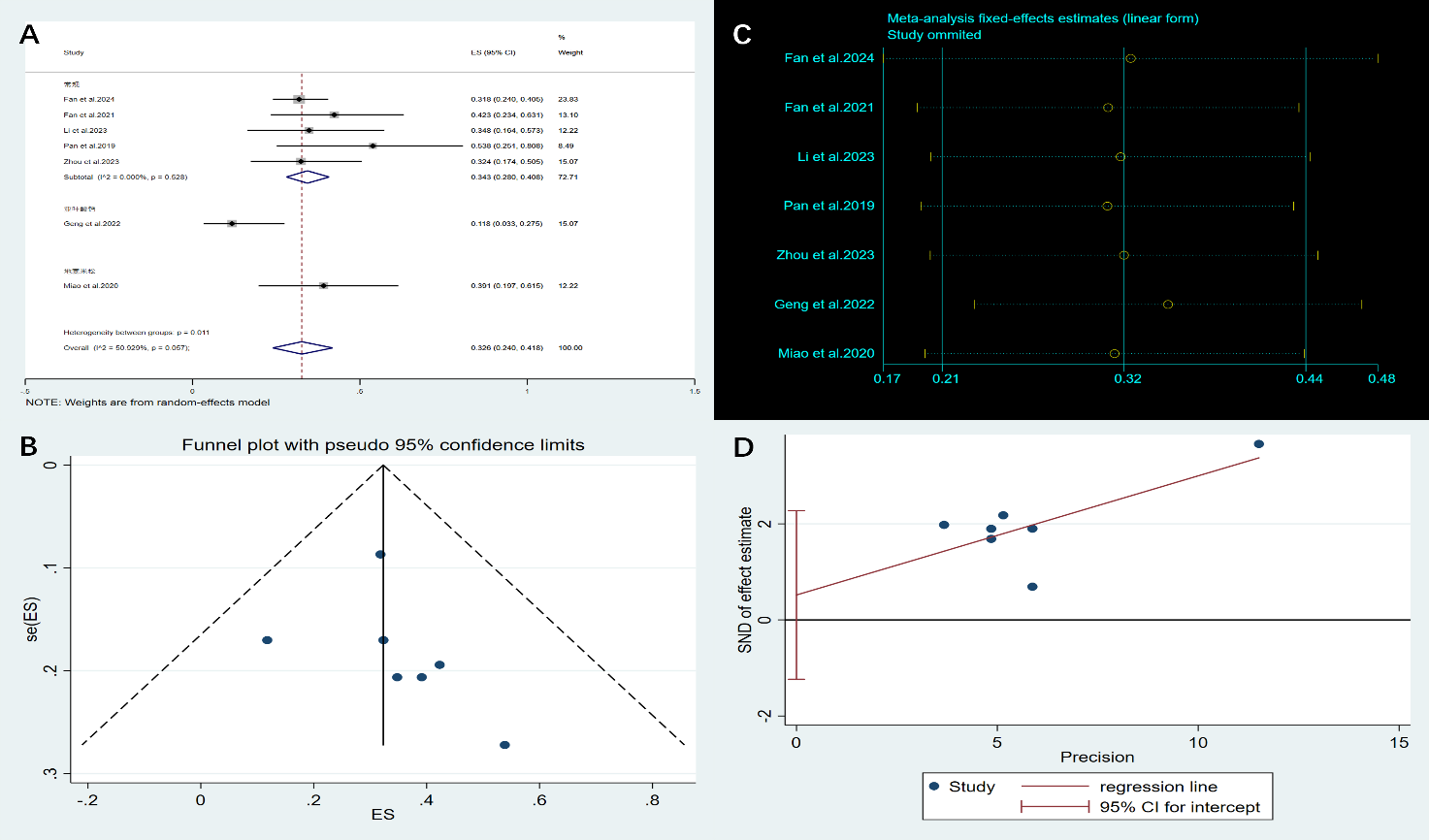


E
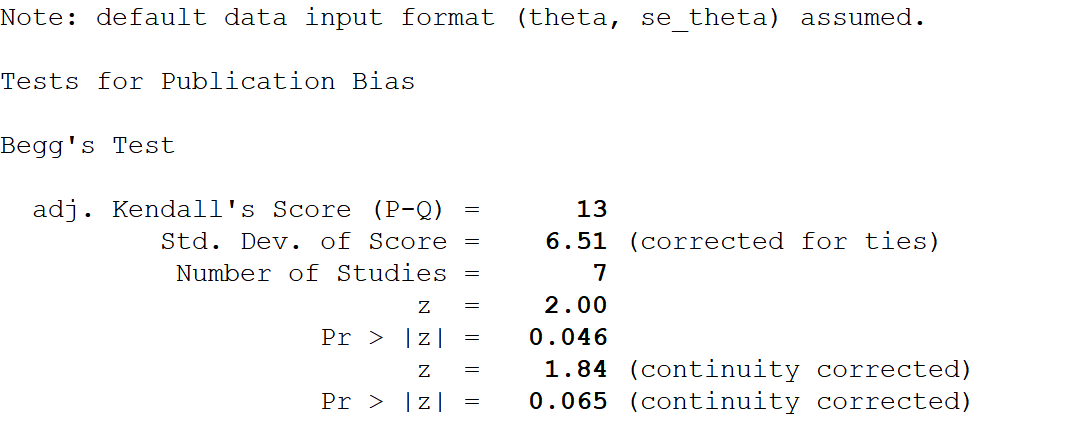


Figure S20 and S21. Subgroup analysis of AEs (All grades): (A) forest plot, (B) sensitivity analysis, (C) funnel plot, (D) Egger's test, (E) Begg's test


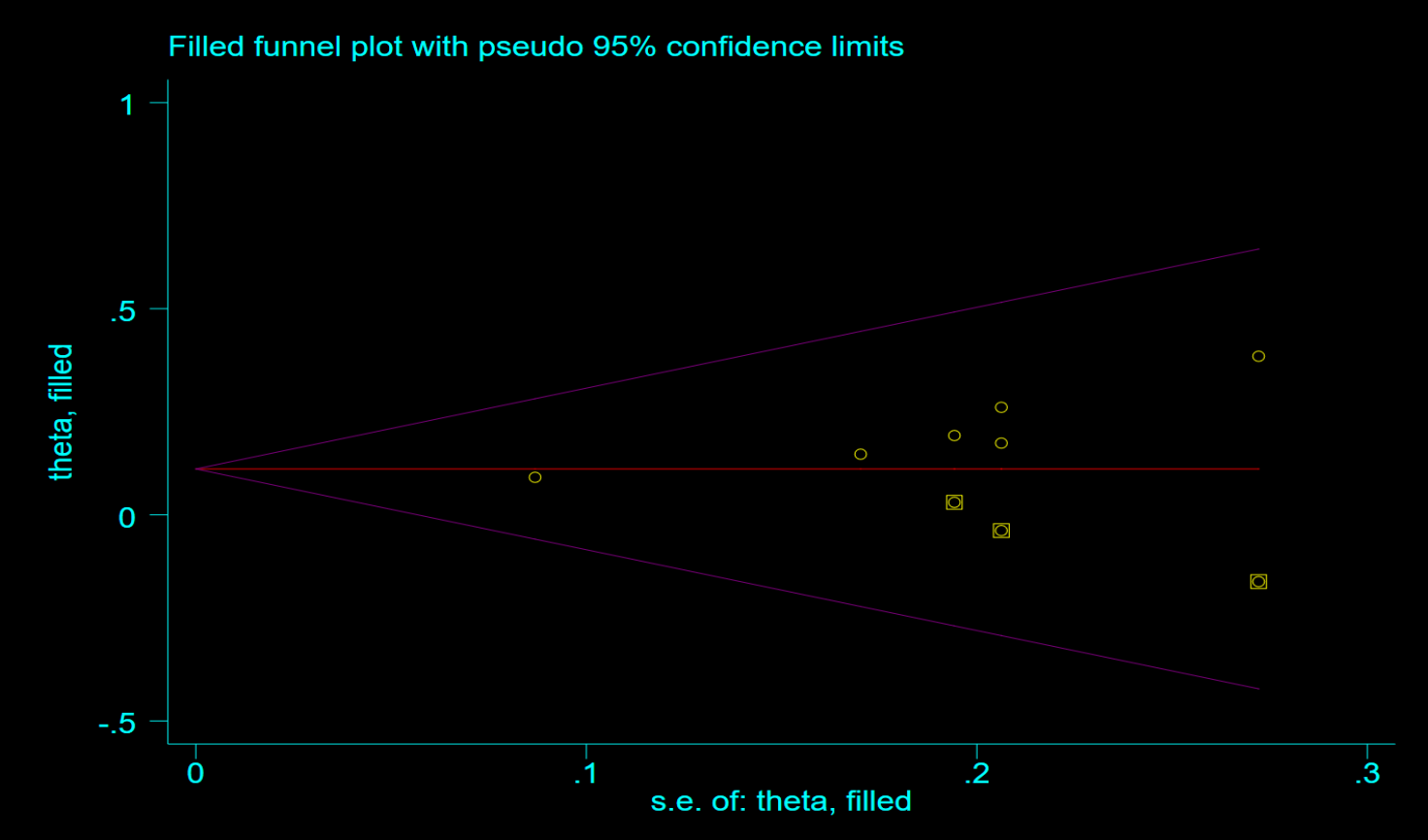


Figure S22. Funnel chart by AEs (All grades) meta-trim
